# Supplementary material for: Cortical and white matter myelination proceed in concert during early infancy
Source: Nat Commun. 2026 Jun 17;17:5353. doi: 10.1038/s41467-026-73366-9 (PMC13276173; doi:10.1038/s41467-026-73366-9)
Supplement: Supplementary file 1 — Supplementary Information [file 41467_2026_73366_MOESM1_ESM.pdf]

Supplementary Information

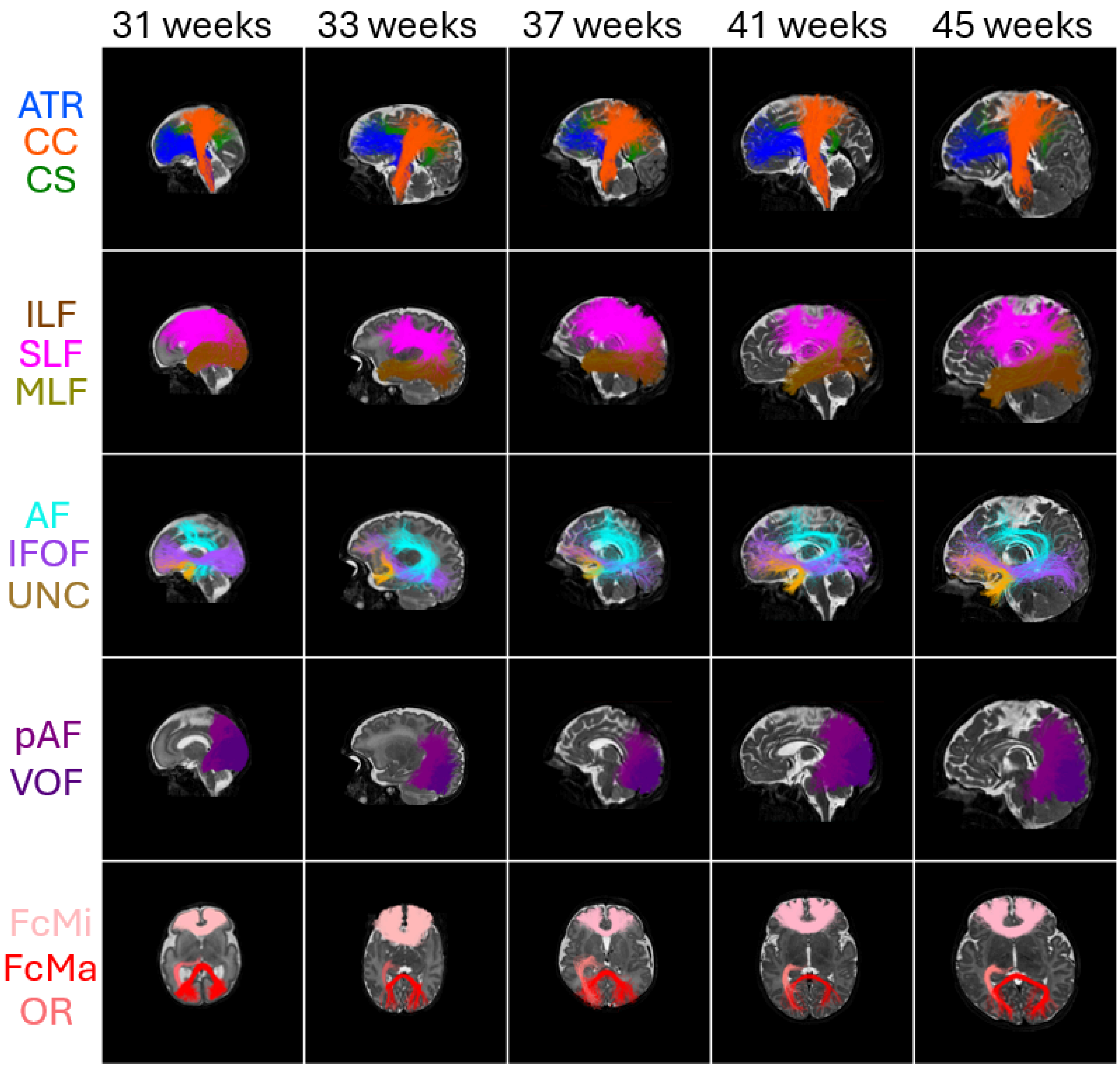

Figure S1: **Examples of bundles identified with pyBabyAFQ in the dHCP dataset.** Bundles are shown in nine randomly chosen individuals from dHCP scanned at postmenstrual ages ranging from 31 weeks (left) to 45 weeks (right). Abbreviations: AF: arcuate fasciculus, ATR: anterior thalamic radiation, CS: cortico-spinal tract, CC: cingulum cingulate, FcMi: forceps minor, FcMa: forceps major, ILF: inferior longitudinal fasciculus, MLF: middle longitudinal fasciculus, IFOF: inferior frontal occipital fasciculus, OR: optic radiation, pAF: posterior arcuate fasciculus, SLF: superior longitudinal fasciculus, UNC: uncinate fasciculus, VOF: ventral occipital fasciculus.

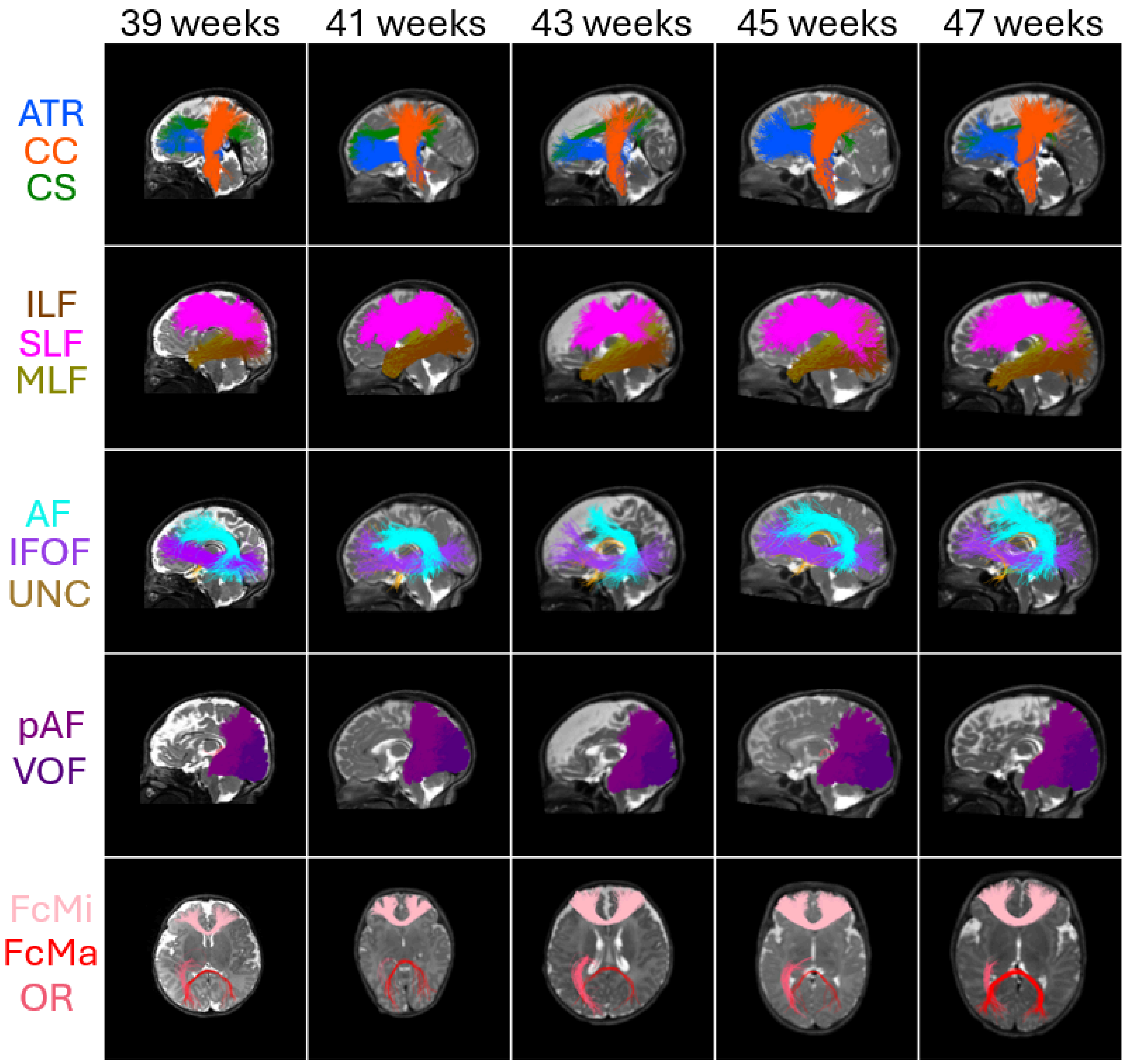

Figures S2: **Examples of bundles identified with pyBabyAFQ in the SVBP dataset.** Bundles are shown in five randomly chosen individuals from SVBP scanned at ages ranging from 39 weeks (left) to 47 weeks (right). Abbreviations: AF: arcuate fasciculus, ATR: anterior thalamic radiation, CS: cortico-spinal tract, CC: cingulum cingulate, FcMi: forceps minor, FcMa: forceps major, ILF: inferior longitudinal fasciculus, MLF: middle longitudinal fasciculus, IFOF: inferior frontal occipital fasciculus, OR: optic radiation, pAF: posterior arcuate fasciculus, SLF: superior longitudinal fasciculus, UNC: uncinate fasciculus, VOF: ventral occipital fasciculus.

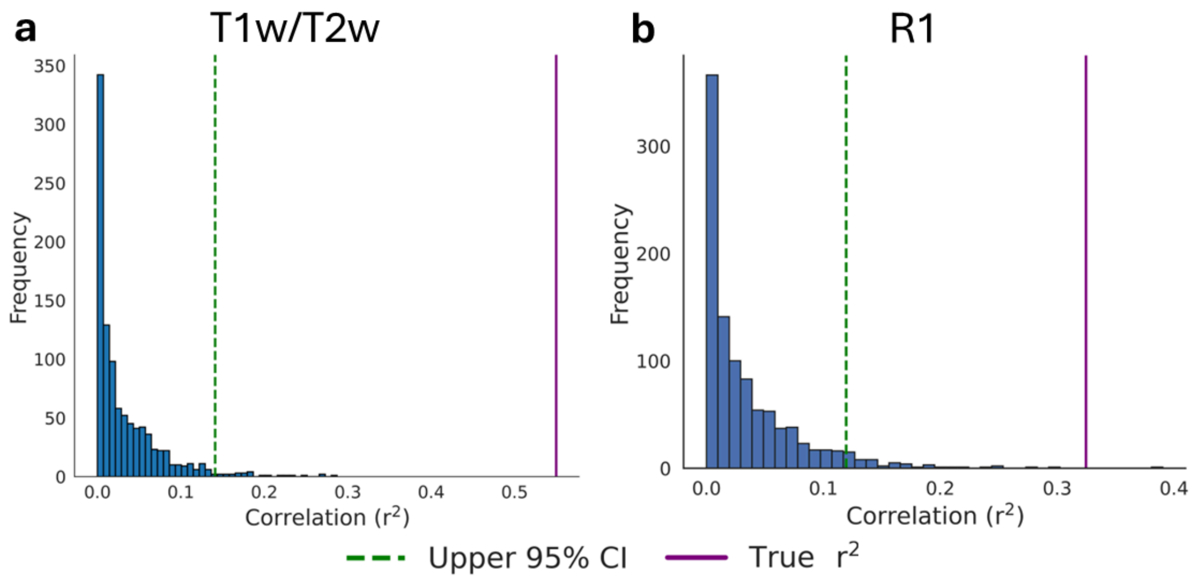

Figure S3: **Results of control analysis.** For this we shuffled bundle–target pairings to estimate chance-level correlations of mean T1w/T2w (a) and R1 (b) values across tissues. The histogram depicts chance correlations observed for 1000 iterations of shuffled bundle–target pairings that omitted the true pairings. The vertical dashed green line indicates the one-sided 95% upper bound of chance-level correlations, and the purple line indicates the observed correlation for the true bundle-target pairings. In both T1w/T2w and R1 the correlations for the true bundle-target pairings are above the 95% one-sided bound.

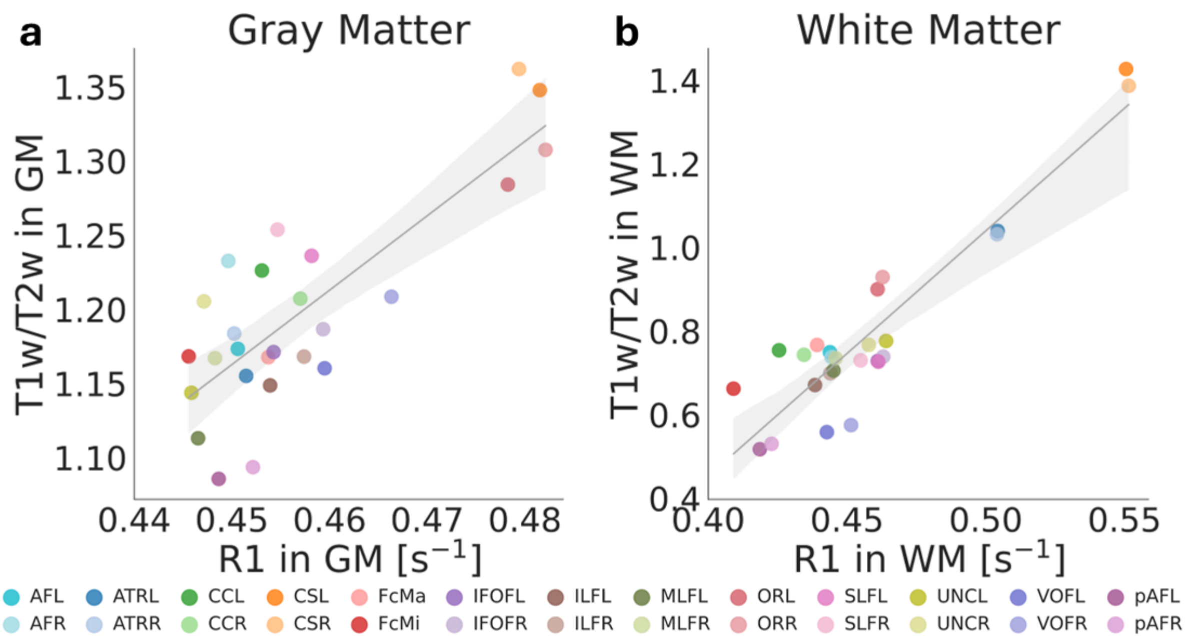

Figure S4: **Relationship between T1w/T2w and R1. a** In gray matter and **b** in white matter. In both tissues the two myelin-sensitive imaging metrics are significantly correlated across bundles (T1w/T2w N=273; R1 N=21; GM: r^2=0.66, p-value=5.32e^{-7}, two-sided; WM: r^2=0.85, r(24)=0.92, p-value=1.88e^{-11}, two-sided). Values are averaged across subjects, and each dot is a bundle, shaded regions indicate 95% confidence interval. Abbreviations: GM: gray matter, WM: white matter, AF: Arcuate Fasciculus, ATR: Anterior Thalamic Radiation], CC: Cingulum Cingulate, CS: Cortico-Spinal Tract, FcMa: Forceps Major, FcMi: Forceps Minor, IFO: Inferior Frontal Occipital Fasciculus, ILF: Inferior Longitudinal Fasciculus, MLF: Middle Longitudinal Fasciculus, OR: Optic Radiation, SLF: Superior Longitudinal Fasciculus, UNC: Uncinate Fasciculus, VOF: Ventral Occipital Fasciculus, pAF: Posterior Arcuate Fasciculus, L=left, R=right.

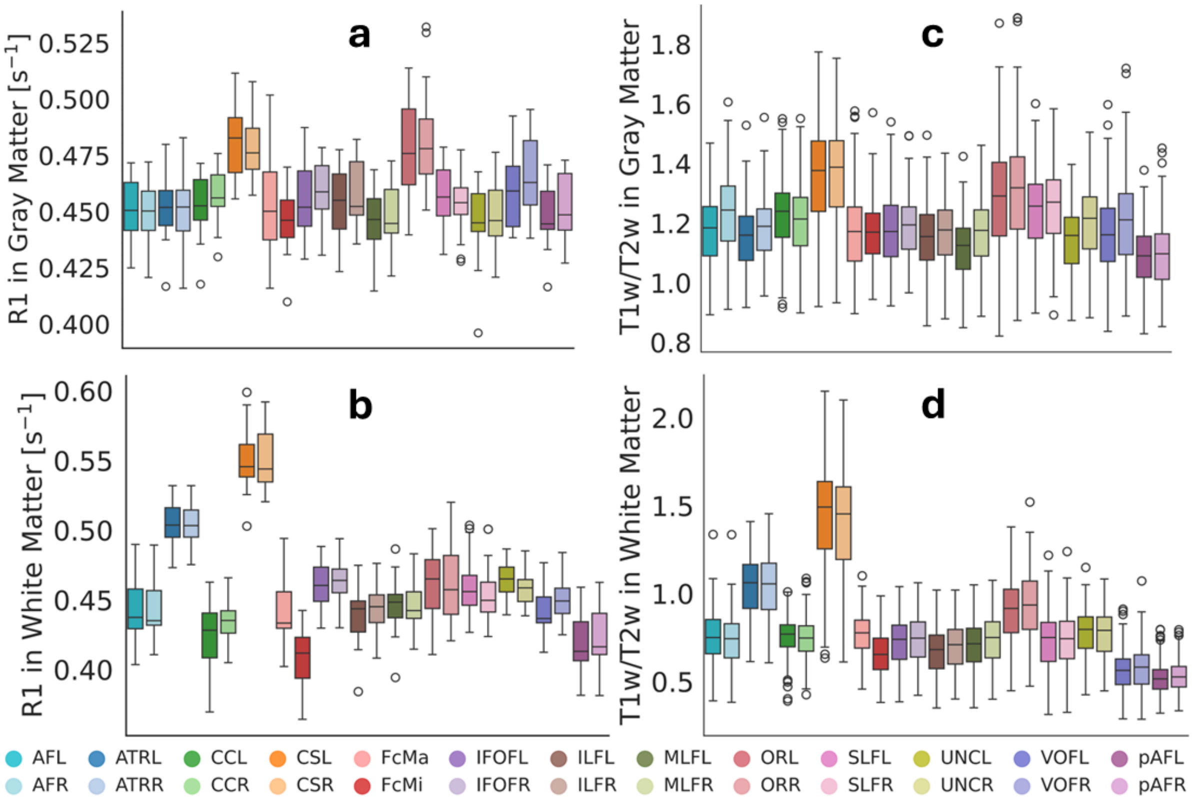

Figure S5: **Boxplots showing differences in T1w/T2w and R1 in white and gray matter across bundles. a,b** R1 (N=21) in gray matter (a) and white matter (b), respectively. **c,d** T1w/T2w (N=273) in gray matter (c) and white matter (d) respectively. Each box represents the interquartile range with median (horizontal line), whiskers indicating 1.5 times the interquartile range, and points representing outliers. Colors indicate bundles. Abbreviations: AF: Arcuate Fasciculus, ATR: Anterior Thalamic Radiation], CC: Cingulum Cingulate, CS: Cortico-Spinal Tract, FcMa: Forceps Major, FcMi: Forceps Minor, IFO: Inferior Frontal Occipital Fasciculus, ILF: Inferior Longitudinal Fasciculus, MLF: Middle Longitudinal Fasciculus, OR: Optic Radiation, SLF: Superior Longitudinal Fasciculus, UNC: Uncinate Fasciculus, VOF: Ventral Occipital Fasciculus, pAF: Posterior Arcuate Fasciculus, L=left, R=right.

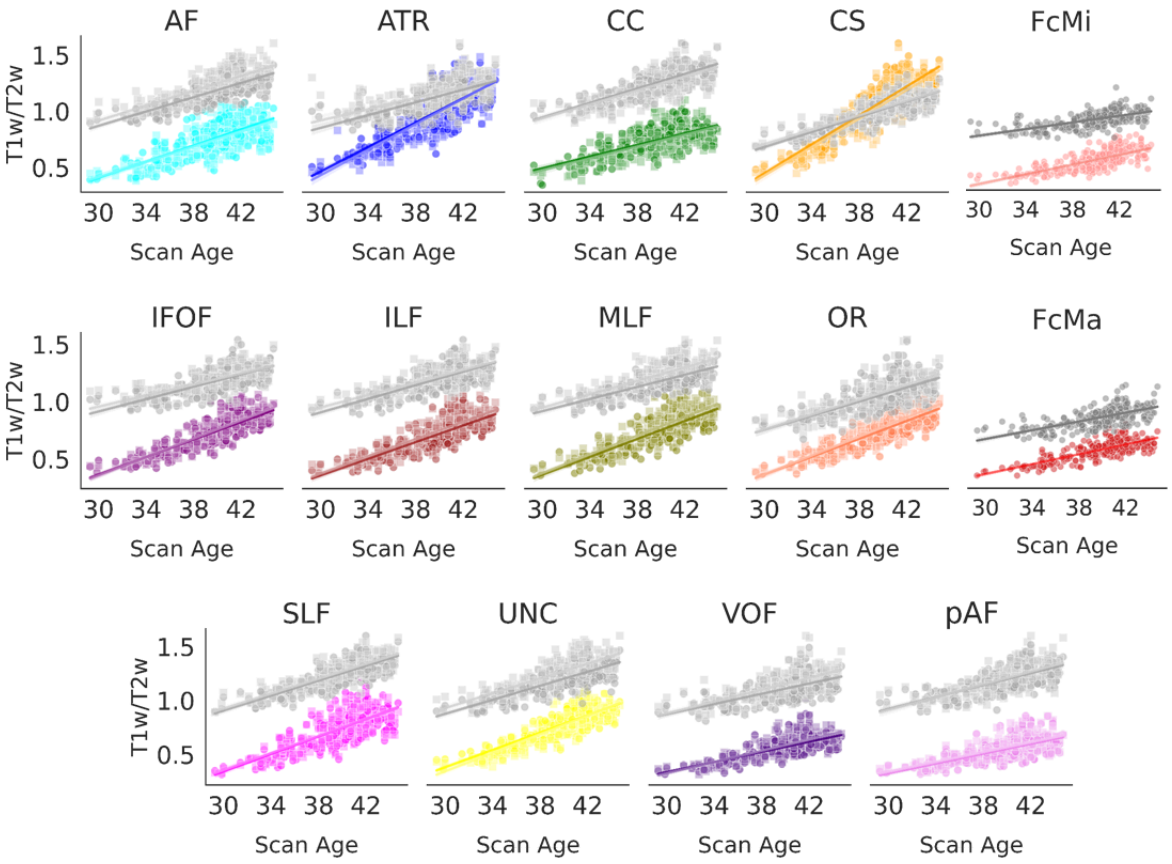

Figure S6: **The rate of change of white and gray matter T1w/T2w across all of the bundles identified with pyBabyAFQ.** Gray markers indicate the correlation for gray matter T1w/T2w (N=273), while colored markers represent the correlation for white matter T1w/T2w (N=273). Opacity indicates the hemisphere, with lower opacity representing the right hemisphere and higher opacity the left hemisphere. The steepness of the lines indicate slopes of T1w/T2w change with increasing postmenstrual age at measurement (in weeks) in each tissue. Shaded regions indicate 95% confidence intervals. Abbreviations: AF: Arcuate Fasciculus, ATR: Anterior Thalamic Radiation], CC: Cingulum Cingulate, CS: Cortico-Spinal Tract, FcMa: Forceps Major, FcMi: Forceps Minor, IFO: Inferior Frontal Occipital Fasciculus, ILF: Inferior Longitudinal Fasciculus, MLF: Middle Longitudinal Fasciculus, OR: Optic Radiation, SLF: Superior Longitudinal Fasciculus, UNC: Uncinate Fasciculus, VOF: Ventral Occipital Fasciculus, pAF: Posterior Arcuate Fasciculus, L=left, R=right.

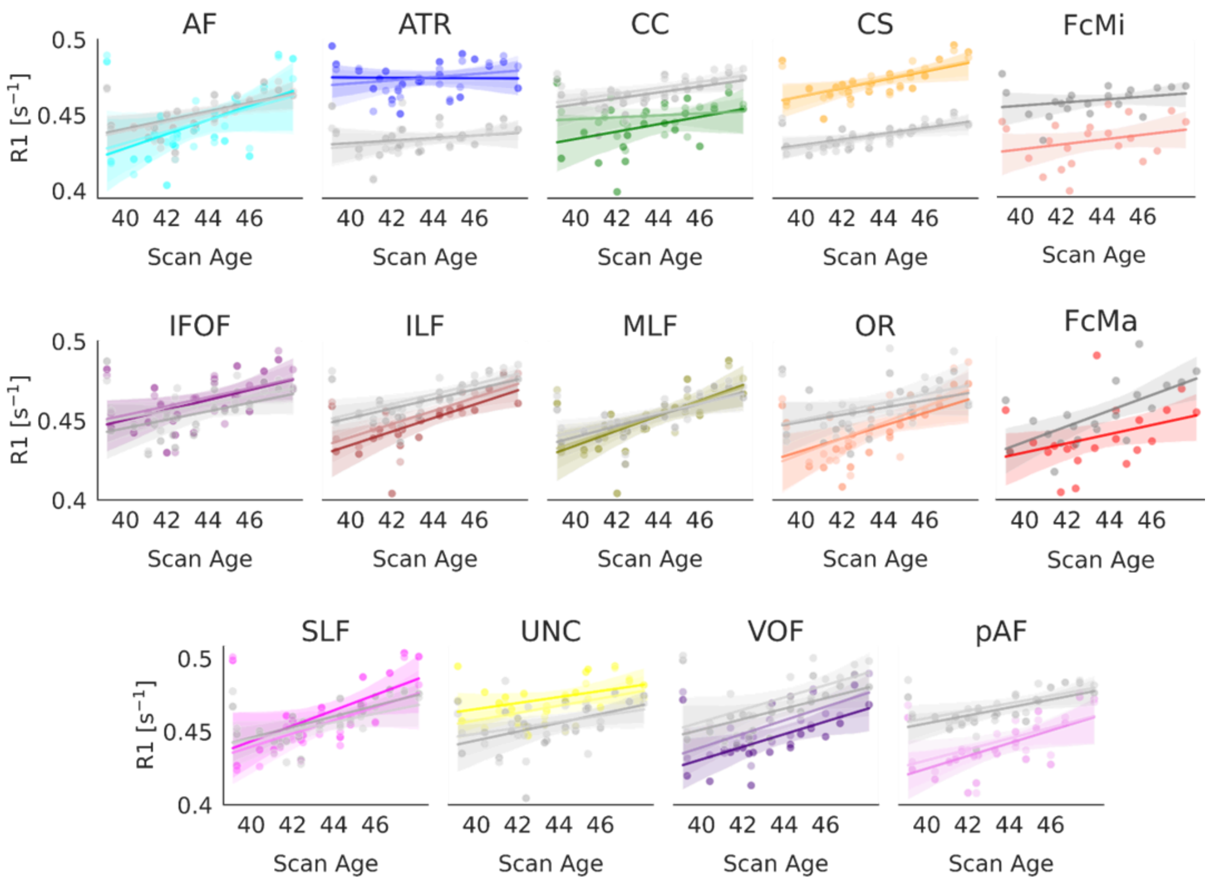

Figure S7: **The rate of change of white and gray matter R1 across all of the bundles identified with pyBabyAFQ.** Gray markers indicate the correlation for gray matter R1 (N=21), while colored markers represent the correlation for white matter R1 (N=21). Opacity indicates the hemisphere, with lower opacity representing the right hemisphere and higher opacity the left hemisphere. The steepness of the lines indicate slopes of R1 change with increasing age at measurement (in weeks) in each tissue. Shaded regions indicate 95% confidence interval. Abbreviations: AF: Arcuate Fasciculus, ATR: Anterior Thalamic Radiation], CC: Cingulum Cingulate, CS: Cortico Spinal Tract, FcMa: Forceps Major, FcMi: Forceps Minor, IFO: Inferior Frontal Occipital Fasciculus, ILF: Inferior Longitudinal Fasciculus, MLF: Middle Longitudinal Fasciculus, OR: Optic Radiation, SLF: Superior Longitudinal Fasciculus, UNC: Uncinate Fasciculus, VOF: Ventral Occipital Fasciculus, pAF: Posterior Arcuate Fasciculus, L=left, R=right.

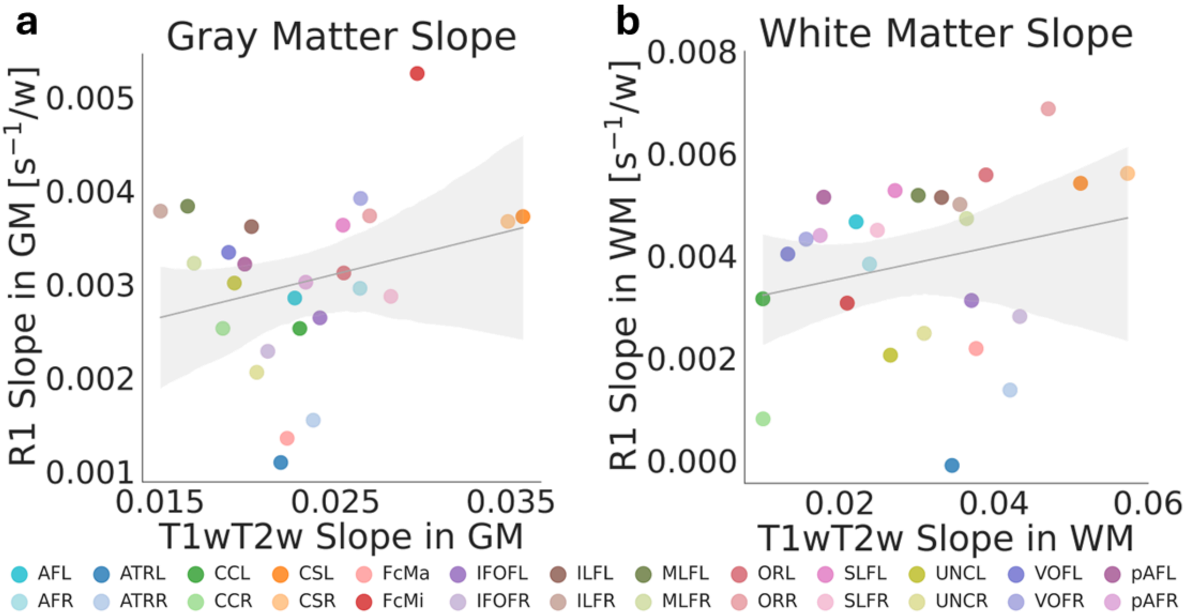

Figure S8: **Relationship between T1w/T2w and R1 developmental slopes in gray matter (a) and white matter (b).** In both tissues the developmental slopes of the two myelin-sensitive imaging metrics are not significantly correlated (GM: r^2=0.07, p-value=0.20, two-sided; WM: r^2=0.06, p-value=0.24, two-sided). The slope indicates the increase in T1w/T2w and R1 (N=21) values relative to the infants' age at the time of measurement in weeks; each dot is a bundle. Shaded regions indicate 95% confidence intervals. Note that we only included those subjects from the dHCP that were within the same age range as the subject of the SVBP (N=183) to ensure that slopes are computed over the same developmental period across metrics. Abbreviations: GM: gray matter, WM: white matter, AF: Arcuate Fasciculus, ATR: Anterior Thalamic Radiation], CC: Cingulum Cingulate, CS: Cortico-Spinal Tract, FcMa: Forceps Major, FcMi: Forceps Minor, IFO: Inferior Frontal Occipital Fasciculus, ILF: Inferior Longitudinal Fasciculus, MLF: Middle Longitudinal Fasciculus, OR: Optic Radiation, SLF: Superior Longitudinal Fasciculus, UNC: Uncinate Fasciculus, VOF: Ventral Occipital Fasciculus, pAF: Posterior Arcuate Fasciculus, L=left, R=right.

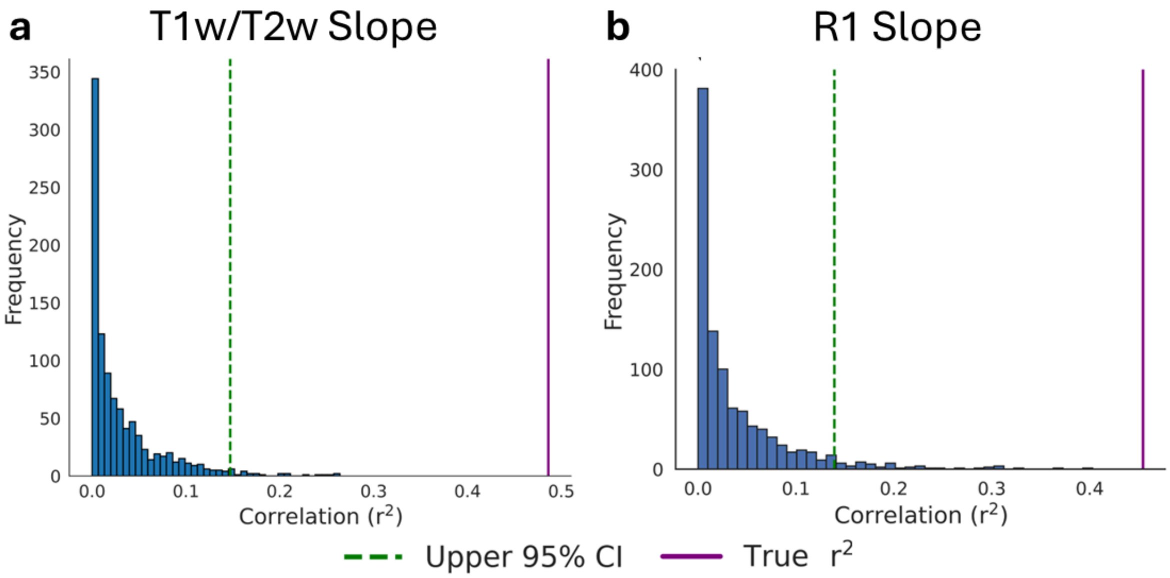

Figure S9: **Results of control analysis for slopes.** For this shuffled bundle–target pairings to estimate chance-level correlations of the slopes of T1w/T2w (a) and R1 (b) development across tissues. The histogram depicts chance correlations observed for 1000 iterations of shuffled bundle–target pairings that omitted the true pairings. The vertical dashed green line indicates the one-sided 95% upper bound of chance-level correlations, and the purple line indicates the observed correlation for the true bundle-target pairings. In both T1w/T2w and R1 the correlations for the true bundle-target pairings are above the 95% one-sided bound.

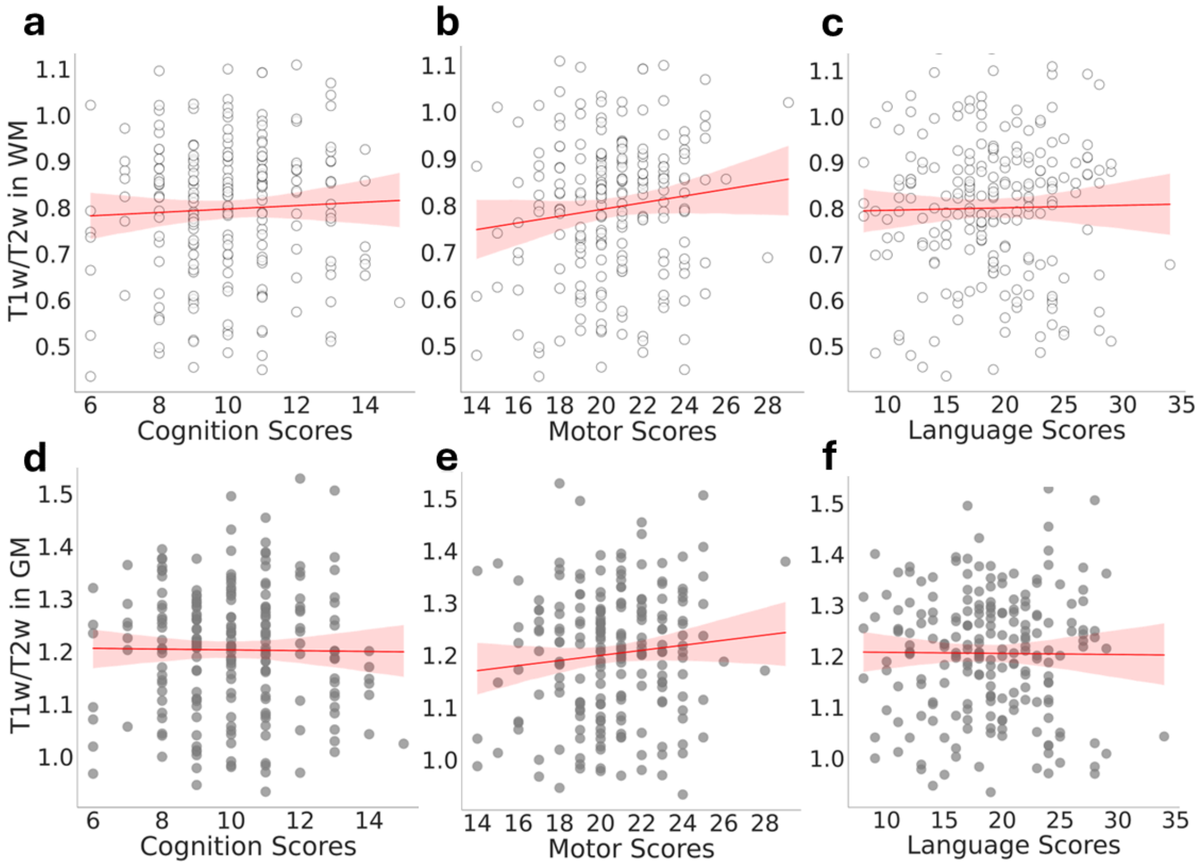

Figure S10: **Relationship between T1w/T2w and cognition, motor, and language performance. a-c** Relationship between T1w/T2w in the white matter and cognition (a; r^2=0.002, p-value=0.50, two-sided), motor (b; r^2=0.02, p-value=0.08, two-sided) and language (c; r^2=0.0003, p-value=0.79, two-sided) subscales of the Bayley-III. **d-f** Relationship between T1w/T2w in the gray matter and cognition (d; N=209; r^2=-0.0001, p-value=0.87, two-sided), motor (e; N=205; r^2=0.01, p-value=0.14, two-sided), and language (f; N=210; r^2=0.0008, p-value=0.89, two-sided) subscales of the Bayley-III. Shaded regions indicate 95% confidence intervals.

T1w/T2w

Bundle	WM p	WM r^2	WM coeff	GM p	GM r^2	GM coeff
AFL	3.26E-42	0.49601121	0.03588858	1.01E-62	0.64396946	0.03183006
AFR	5.88E-42	0.49382503	0.0341056	4.83E-56	0.60128482	0.03317838
ATRL	4.40E-66	0.66370581	4.48E-02	2.18E-38	0.46230977	0.02336754
ATRR	7.29E-67	0.66813018	0.04688158	7.72E-30	0.3786821	0.02065157
CCL	7.79E-40	0.47531305	0.02387939	2.94E-61	0.63502846	0.02978607
CCR	5.44E-32	0.40086905	0.02437258	2.42E-46	0.53004997	0.02844867
CSL	1.24E-88	0.77062602	0.08577072	1.77E-74	0.7083524	0.04492214
CSR	5.20E-84	0.751909	0.08429417	2.71E-67	0.67053776	0.04286874
FcMi	5.04E-48	0.54325362	0.03047732	7.63E-31	0.38914321	0.02712702
FcMa	3.30E-56	0.6024077	0.03014956	3.46E-36	0.4419104	0.02049164
IFOFL	4.83E-69	0.68017531	0.03820163	2.13E-44	0.51432327	0.02698286
IFOFR	3.35E-74	0.70697334	0.03996037	6.77E-42	0.49330271	0.02417439
ILFL	2.92E-62	0.64118653	0.03568442	1.29E-50	0.56288097	0.02914318
ILFR	1.86E-66	0.66583242	0.03703395	1.13E-47	0.54052215	0.02678297
MLFL	2.91E-62	0.64119827	0.03665657	3.90E-50	0.55930199	0.02484381
MLFR	1.44E-68	0.67759523	0.03846351	4.64E-49	0.55119713	0.02753962
ORL	1.59E-66	0.66622128	0.04810809	5.59E-38	0.45857353	0.03797427
ORR	1.15E-65	0.6613197	0.05121672	2.41E-40	0.47981063	0.04007292
SLFL	2.46E-52	0.57543018	0.04031047	6.45E-66	0.66275742	0.03468034
SLFR	1.30E-51	0.57020614	0.03956626	4.15E-64	0.65225524	0.03460147
UNCL	2.67E-71	0.69218966	0.03503019	1.19E-58	0.61852777	0.02833703
UNCR	7.06E-80	0.73388298	0.03747226	1.58E-51	0.56957773	0.02993709
VOFL	1.12E-45	0.52473566	0.02609958	5.36E-34	0.42084628	0.02645496
VOFR	1.13E-48	0.54823631	0.02909089	1.70E-39	0.47228797	0.03073249
pAFL	3.70E-39	0.46926914	0.02012696	1.02E-44	0.51695343	0.0244694
pAFR	3.09E-38	0.46092024	0.02004629	1.14E-44	0.51655634	0.02606878

Table S1: **Relationship between postmenstrual age and T1w/T2w in each white matter bundle (WM) and their gray matter (GM) targets.** The table summarizes the statistical relationships between scan age (postmenstrual age at measurement in weeks) and T1w/T2w in each bundle (N=273). For each bundle, the correlation (r^2), the coefficient (slope), and the associated p-value is reported separately for white matter (WM) and gray matter (GM). All bundles show statistically significant correlations (all ps < 0.05), as highlighted in green. Abbreviations: GM: gray matter, WM: white matter, AF: Arcuate Fasciculus, ATR: Anterior Thalamic Radiation], CC: Cingulum Cingulate, CS: Cortico-Spinal Tract, FcMa: Forceps Major, FcMi: Forceps Minor, IFO: Inferior Frontal Occipital Fasciculus, ILF: Inferior Longitudinal Fasciculus, MLF: Middle Longitudinal Fasciculus, OR: Optic Radiation, SLF: Superior Longitudinal Fasciculus, UNC: Uncinate Fasciculus, VOF: Ventral Occipital Fasciculus, pAF: Posterior Arcuate Fasciculus, L=left, R=right.

R1

Bundle	WM p	WM r^2	WM coeff	GM p	GM r^2	GM coeff
AFL	0.02197953	0.24675132	0.00466576	0.00649369	0.32962908	0.00285392
AFR	0.04100023	0.20190967	0.00383947	0.00934692	0.30560913	0.00295793
ATRL	0.94401978	0.00026639	-9.69E-05	0.36799227	0.0428421	0.00109592
ATRR	0.30297424	0.05571395	0.0013761	0.20932102	0.08162375	0.00154812
CCL	0.12518794	0.11927374	0.00315954	0.01885071	0.25756295	0.00252821
CCR	0.54563896	0.01953842	0.00081258	0.01501227	0.27340477	0.00252928
CSL	0.00349122	0.36903122	0.00541797	0.00162504	0.41490455	0.00372508
CSR	0.00060239	0.47006738	0.00561202	0.00269811	0.38482106	0.00367237
FcMi	0.24106061	0.07156173	0.00307622	0.25536165	0.06750817	0.00525478
FcMa	0.10296567	0.13379251	0.00218789	0.00173945	0.41094313	0.00135454
IFOFL	0.0268136	0.23261049	0.00312842	0.04238307	0.19948637	0.00264276
IFOFR	0.01881613	0.25769162	0.00281515	0.03487151	0.21368809	0.00228502
ILFL	0.00148988	0.41992715	0.00514167	0.00211819	0.39934153	0.00361781
ILFR	0.00011579	0.55135401	0.00500508	0.00095596	0.44500272	0.00378354
MLFL	0.00132246	0.42675957	0.00518177	0.00054973	0.47490963	0.00383553
MLFR	0.00077035	0.4568491	0.00472878	0.00243026	0.39113109	0.0032256
ORL	0.00748707	0.32031908	0.00558123	0.10497975	0.13235144	0.00312207
ORR	0.00216663	0.39799733	0.00687402	0.05384594	0.18190798	0.00373258
SLFL	0.00444538	0.35392143	0.00527617	0.000531	0.47673339	0.0036329
SLFR	0.01465996	0.27504373	0.00449986	0.01806566	0.26054001	0.00287125
UNCL	0.10039371	0.1356746	0.00205944	0.03977016	0.20413202	0.00301418
UNCR	0.01978177	0.25417942	0.00248636	0.12265305	0.12079099	0.00205944
VOFL	0.00753326	0.31991458	0.00403512	0.01647233	0.26697471	0.00334213
VOFR	0.00179929	0.40896507	0.00432513	0.00824688	0.31394034	0.00391899
pAFL	0.00363345	0.36655449	0.00514678	0.00389854	0.36216688	0.00321604
pAFR	0.00976442	0.30268367	0.00439779	0.008136	0.31483634	0.00302265

Table S2: **Relationship between postmenstrual age and R1 in each white matter bundle (WM) and their gray matter (GM) targets.** The table summarizes the statistical relationships between scan age (postmenstrual age at measurement in weeks) and R1 in each bundle (N=21). For each bundle, the correlation (r^2), the coefficient (slope), and the associated p-value is reported separately for white matter (WM) and gray matter (GM). Statistically significant correlations ($p < 0.05$) are highlighted in green. Abbreviations: GM: gray matter, WM: white matter, AF: Arcuate Fasciculus, ATR: Anterior Thalamic Radiation], CC: Cingulum Cingulate, CS: Cortico-Spinal Tract, FcMa: Forceps Major, FcMi: Forceps Minor, IFO: Inferior Frontal Occipital Fasciculus, ILF: Inferior Longitudinal Fasciculus, MLF: Middle Longitudinal Fasciculus, OR: Optic Radiation, SLF: Superior Longitudinal Fasciculus, UNC: Uncinate Fasciculus, VOF: Ventral Occipital Fasciculus, pAF: Posterior Arcuate Fasciculus, L=left, R=right.
