# Supplementary material for: Cortical and white matter myelination proceed in concert during early infancy
Source: Nat Commun. 2026 Jun 17;17:5353. doi: 10.1038/s41467-026-73366-9 (PMC13276173; doi:10.1038/s41467-026-73366-9)
Supplement: Supplementary file 2 — Reporting Summary [file 41467_2026_73366_MOESM2_ESM.pdf]

nature portfolio

Corresponding author(s): Zika, Stephanie

Last updated by author(s): Mar 26, 2026

Reporting Summary

Nature Portfolio wishes to improve the reproducibility of the work that we publish. This form provides structure for consistency and transparency in reporting. For further information on Nature Portfolio policies, see our Editorial Policies and the Editorial Policy Checklist.

Statistics

For all statistical analyses, confirm that the following items are present in the figure legend, table legend, main text, or Methods section.

n/a	Confirmed	
☐	☒	The exact sample size (n) for each experimental group/condition, given as a discrete number and unit of measurement
☐	☒	A statement on whether measurements were taken from distinct samples or whether the same sample was measured repeatedly
☐	☒	The statistical test(s) used AND whether they are one- or two-sided<br>*Only common tests should be described solely by name; describe more complex techniques in the Methods section.*
☐	☒	A description of all covariates tested
☐	☒	A description of any assumptions or corrections, such as tests of normality and adjustment for multiple comparisons
☐	☒	A full description of the statistical parameters including central tendency (e.g. means) or other basic estimates (e.g. regression coefficient) AND variation (e.g. standard deviation) or associated estimates of uncertainty (e.g. confidence intervals)
☐	☒	For null hypothesis testing, the test statistic (e.g. F, t, r) with confidence intervals, effect sizes, degrees of freedom and P value noted<br>*Give P values as exact values whenever suitable.*
☒	☐	For Bayesian analysis, information on the choice of priors and Markov chain Monte Carlo settings
☒	☐	For hierarchical and complex designs, identification of the appropriate level for tests and full reporting of outcomes
☐	☒	Estimates of effect sizes (e.g. Cohen's d, Pearson's r), indicating how they were calculated

Our web collection on statistics for biologists contains articles on many of the points above.

Software and code

Policy information about availability of computer code

Data collection	No software was used for data collection
Data analysis	The data were analyzed using open-source software, including iBEAT V2.0 (https://ibeat.wildapricot.org/), ITKgray, MRtrix3 (RC3, https://www.mrtrix.org/), FreeSurfer 7.4.1 (https://surfer.nmr.mgh.harvard.edu/fswiki/FreeSurferWiki), FSL (v6.0.2, https://fsl.fmrib.ox.ac.uk/) and pyBabyAFQ, which we shared as a component of pyAFQ (https://yeatmanlab.github.io/pyAFQ). Cloudknot software was used to deploy both tractography and bundle identification to the Amazon Web Services (AWS) Batch service. Code that implemented the tractography pipeline, example code to perform bundle identification with pyBabyAFQ, and code used to generate the main figures of this manuscript are also made available on GitHub (https://github.com/EduNeuroLab/WMGMMyelinInfants).

For manuscripts utilizing custom algorithms or software that are central to the research but not yet described in published literature, software must be made available to editors and reviewers. We strongly encourage code deposition in a community repository (e.g. GitHub). See the Nature Portfolio guidelines for submitting code & software for further information.

Data

Policy information about availability of data

All manuscripts must include a data availability statement. This statement should provide the following information, where applicable:

- Accession codes, unique identifiers, or web links for publicly available datasets
- A description of any restrictions on data availability
- For clinical datasets or third party data, please ensure that the statement adheres to our policy

All data required to generate the main figures are made available in GitHub (https://github.com/EduNeuroLab/WMGMMyelinInfants). Source Data are provided with this paper.

Research involving human participants, their data, or biological material

Policy information about studies with human participants or human data. See also policy information about sex, gender (identity/presentation), and sexual orientation and race, ethnicity and racism.

Reporting on sex and gender	We are using openly available data from the Developing Human Connectome Project (dHCP) and the locally collected data from Stanford University (Stanford VPNL Baby Project (SVBP)). We report information on infant sex (male/female), which is typically assigned at birth or inferred from medical records. As the participants are infants, gender identity cannot be known or meaningfully assessed at this developmental stage.
Reporting on race, ethnicity, or other socially relevant groupings	This study uses data from the Developing Human Connectome Project (dHCP), which included demographic information collected through maternal self-report. Mothers of participating infants completed a questionnaire in which they selected their self-identified ethinicity ethnicity according to United Kingdom census categories and is included in the participant section within the methods. Race and ethnicity: 94 White British, 53 White Other, 38 Black/Black British - African, 17 Chinese, 14 Other, 13 Asian/Asian British - Indian, 7 Asian/Asian British - Other, 7 Black/Black British - Caribbean, 6 Any Other Mixed Ethnic Group, 6 White Irish, 6 Unknown, 3 Asian/Asian British - Bangladeshi, 3 Asian/Asian British - Pakistani, 2 White and Black Caribbean, 2 White And Asian, 1 White and Black African, 1 Black/Black British - Other participants<br><br>For SVBP data, demographic information was also collected through maternal report and is included in the participant section within the methods: Race and ethnicity: 3 Asian, 3 Hispanic, 5 Multiracial, and 10 White participants.
Population characteristics	dHCP:<br>The data comprised 311 sessions from 273 individuals, including both preterm (gestational age < 37 weeks) and full-term born infants. Of these, 117 were female. The gestational age at birth ranged from 25.57 to 42.29 weeks (mean ± SD: 38.05 ± 3.67 weeks). Scans were performed between 29.29 and 44.71 weeks post-conceptional age (mean ± SD: 39.53 ± 3.11 weeks), with the time interval between birth and scan ranging from 0 to 16.42 weeks (mean ± SD: 1.48 ± 2.02 weeks).<br><br>SVBP:<br>The data contained 21 infants scanned shortly after birth. Among these participants, 7 were female. The gestational age at birth ranged from 37 to 42 weeks (mean ± SD: 39.09 ± 1.63 weeks). Imaging was conducted between 39.10 and 48.10 weeks post-conceptional age (mean ± SD: 43.39 ± 2.53 weeks), corresponding to an interval of 2.3 to 7.1 weeks after birth (mean ± SD: 4.30 ± 1.35 weeks). Race and ethnicity: 3 Asian, 3 Hispanic, 5 Multiracial, and 10 White participants.
Recruitment	dHCP:<br>According th the dHCP, recruitment was done by at St Thomas' Hospital, London and imaging took place at the Evelina Newborn Imaging Centre, Centre for the Developing Brain, King's College London, United Kingdom. Term-born infants were recruited from the postnatal wards and approached on the basis of being clinically well. Preterm-born infants were recruited from the neonatal unit and postnatal wards. Infants were not approached for study inclusion if there was a history of severe compromise at birth requiring prolonged resuscitation, a diagnosed chromosomal abnormality or any contraindication to MRI scanning (e.g. due to incompatible implants). No infants included in the final study group required treatment for clinically significant brain injury. Information on participant compensation was not readily attainable.<br><br>SVBP:<br>Expectant mothers and their infants were recruited from the San Francisco Bay Area using social media platforms. A two-step screening process for expectant mothers was performed. First, mothers were screened over the phone for eligibility based on exclusionary criteria designed to recruit a sample of typically developing infants and second, eligible expectant mothers were screened once again after giving birth. Exclusionary criteria for expectant mothers were as follows: recreational drug use during pregnancy, significant alcohol use during pregnancy (more than three instances of alcohol consumption per trimester; more than 1 drink per occasion), lifetime diagnosis of autism spectrum disorder or a disorder involving psychosis or mania, taking prescription medications for any of these disorders during pregnancy, insufficient written and spoken English ability to understand the instructions of the study, or learning disabilities that would preclude participation in the study. Exclusionary criteria for infants were: birth before 36 weeks of gestation, low birthweight (<5 lbs 8 oz), small height (<18 inches), any congenital, genetic, and neurological disorders, visual problems, complications during birth that involved the infant (e.g., NICU stay), history of head trauma, and contraindications for MRI (e.g., metal implants). Study protocols for these scans were approved by the Stanford University Internal Review Board on Human Subjects Research. Participants were compensated with 25 dollars per hour for their participation in the study.
Ethics oversight	According to the dHCP, their study was approved by the United Kingdom Health Research Authority (Research Ethics Committee reference number: 14/LO/1169). Written consent was obtained from all participating families prior to imaging.

Data analyses procedures were approved by the Ethics Board of the medical facility of Marburg University.

For the SVBP, the study was approved by the Institutional Review Board of Stanford University and complies with all ethical regulations. Parents of infant participants provided written informed consent prior to their scan session. Participants were compensated with 25 dollars per hour for their participation in the study.

Note that full information on the approval of the study protocol must also be provided in the manuscript.

Field-specific reporting

Please select the one below that is the best fit for your research. If you are not sure, read the appropriate sections before making your selection.

☐ Life sciences ☒ Behavioural & social sciences ☐ Ecological, evolutionary & environmental sciences

For a reference copy of the document with all sections, see nature.com/documents/nr-reporting-summary-flat.pdf

Behavioural & social sciences study design

All studies must disclose on these points even when the disclosure is negative.

Study description

Quantitative behavioral data

Research sample

The data encompassed 490 sessions, acquired from 445 individuals, which included all necessary data for the current analyses (diffusion MRI, T1-weighted, and T2-weighted images). After quality assurance (described below), the dHCP data comprised 311 sessions from 273 individuals, including both preterm (gestational age < 37 weeks) and full-term born infants. Of these, 117 were female (Race and ethnicity: 94 White British, 53 White Other, 38 Black/Black British - African, 17 Chinese, 14 Other, 13 Asian/Asian British - Indian, 7 Asian/Asian British - Other, 7 Black/Black British - Caribbean, 6 Any Other Mixed Ethnic Group, 6 White Irish, 6 Unknown, 3 Asian/Asian British - Bangladeshi, 3 Asian/Asian British - Pakistani, 2 White and Black Caribbean, 2 White And Asian, 1 White and Black African, 1 Black/Black British - Other participants). The gestational age at birth ranged from 25.57 to 42.29 weeks (mean ± SD: 38.05 ± 3.67 weeks). Scans were performed between 29.29 and 44.71 weeks post-conceptional age (mean ± SD: 39.53 ± 3.11 weeks), with the time interval between birth and scan ranging from 0 to 16.42 weeks (mean ± SD: 1.48 ± 2.02 weeks). In addition to this main cross-sectional sample, we also derived two smaller subsamples: The first subsample included cross-sectional data only from those 215 infants (93 females, 55 infants born preterm) that complete the Bayley-III Scales of Infant and Toddler Development, which was collected between 17 and 25 months of age (mean ± SD: 19.15 ± 1.39 months). The second subsample took advantage of the longitudinal data and consisted of two groups. The first group included 26 preterm infants (8 females) who were scanned twice: once shortly after their preterm birth and again at term-equivalent age. For these infants, the mean gestational age at birth was 32.04 ± 2.97 weeks. The first scan occurred at a mean age of 34.33 ± 1.76 weeks, while the second scan was performed at a mean age of 40.70 ± 1.08 weeks. The second group comprised 26 full-term infants matched 1:1 to the preterm group in terms of sex and age at the second scan. This full-term group included 8 females, with a mean gestational age at birth of 40.03 ± 0.82 weeks and a mean scan age of 40.67 ± 1.07 weeks. The data is representative for London and surrounding area. This dataset was chosen due to the large cohort.

In addition, we also used anatomical, diffusion-weighted and R1 infant data collected locally at Stanford University. We refer to this data as the Stanford VPNL Baby Project (SVBP). The data encompassed 27 sessions, acquired from 27 individuals, which included all necessary data for the current analyses (diffusion MRI, R1 maps, and anatomical data). After quality assurance (described below) the data contained 21 infants scanned shortly after birth (Race and ethnicity: 3 Asian, 3 Hispanic, 5 Multiracial, and 10 White participants). Among these participants, 7 were female. The gestational age at birth ranged from 36.5 to 42 weeks (mean ± SD: 39.09 ± 1.63 weeks). Imaging was conducted between 39.10 and 48.10 weeks post-conceptional age (mean ± SD: 43.39 ± 2.53 weeks), corresponding to an interval of 2.3 to 7.1 weeks after birth (mean ± SD: 4.30 ± 1.35 weeks). Study protocols for these data were approved by the Stanford University Internal Review Board on Human Subjects Research. The data is representative for Stanford and surrounding area and was chosen due to qMRI measurements in infants.

Sampling strategy

This study utilized openly available data from the dHCP as well as locally collected data from the Stanford VPNL Baby Project (SVBP). In both datasets we used all data available on the onset of study, without sample size calculation. Even though the datasets differ in sample sizes, they still show the same results. Therefore the respective sample sizes are sufficient for the aim of our study.

Data collection

dHCP:

All data were acquired on a 3T Philips Achieva with a dedicated neonatal imaging system including a neonatal 32-channel phased-array head coil, sited within the neonatal intensive care unit at the Evelina London Children's Hospital. According to the dHCP documentation, participants were imaged in natural sleep, with six exceptions who were sedated with chloral hydrate. If a baby woke up, scanning was halted and the infant settled without taking them out of the imaging cradle. Infants were imaged following feeding and swaddling in a vacuum-evacuated blanket. Infants were provided with hearing protection in the form of: molded dental putty placed in the external auditory meatus (President Putty, Coltene Whaledent, Mahwah, NJ, United States); Minimuffs (Natus Medical Inc., San Carlos, CA, United States); and an acoustic hood. Monitoring throughout the scanning session (In vivo Expression, Philips, Best, NL), included pulse oximetry, respiration (using a small air cushion placed on the lower abdomen) and body temperature via a fiber optic probe placed in the axilla. The bespoke imaging cradle system placed subjects in a standardized pose and allowed a fixed imaging geometry to be deployed, with only the position in the head-foot direction adjusted at the start of the examination.

To reduce the risk of waking infants due to startle responses at the start of new sequences, the scanner software was modified to ramp up the gradient waveforms gradually over 5 s as each acquisition commenced and prior to any radiofrequency (RF) pulses or data being acquired. Calibration scans, anatomical images (T1w and T2w), resting state functional (rs-fMRI) and diffusion (dMRI) acquisitions were acquired, with an average data rate of 27 slices/second including all preparation and calibration phases. The acquisition protocol was optimized for the properties of the neonatal brain and for efficiency.

	SVBP:<br>Scanning sessions were scheduled in the evenings around infants' bedtime and were done during natural sleep. Infant data were acquired on a 3T GE Ultra High Performance (UHP) scanner (GE550 Healthcare, Waukesha, WI) equipped with a customized 32-channel infant head-coil. Hearing protection included soft wax earplugs, and MRI compatible neonatal noise attenuators (https://newborncare.natus.com/products-services/newborn-care-products/nursery-essentials/minimuffs-neonatal-noise-attenuators), and headphones (https://www.alpinehearingprotection.com/products/muffy-baby) that covered the infant's ears. An MR-safe plastic immobilizer (MedVac, www.supertechx-ray.com) was used to stabilize the infant and their head position. When the infant was asleep, the caregiver placed the infant on the scanner bed. Weighted bags were placed at the edges of the bed to prevent side-to-side movements. Pads were also placed around the infant's head and body to stabilize head position. An experimenter stayed inside the MR suite with the infant during the entire scan. During scan, experimenters monitored the infant using an infrared camera that was affixed to the head coil and positioned for viewing the infant's face. Experimenters stopped the scan if the infant showed signs of waking or distress or excessively moved; scans were repeated if there was excessive head motion.
Timing	dHCP:<br>All imaging data used in this study involved neonatal brain scans aquired continously between 2017 and 2019 and is part of an open-source dataset. We did not collect any data.<br><br>SVBP:<br>Locally collected data used in this study involved neonatal brain scans aquired between 2019 and 2022. Scans were not aquired continously, due to a break in 2020 caused by the covid pandemic.
Data exclusions	dHCP:<br>For the dHCP data we implemented automated quality assurance steps suitable for a large data set. First, we excluded all sessions that exceeded two standard deviations from the mean with respect to absolute motion and the number of outlier slices replaced by FSL's eddy as in our prior work. Eight additional sessions were excluded due to conspicuous image artifacts or alignment errors that were noted during data processing. Further, to ensure bundle quality, we excluded all sessions where one or more white matter bundles contained 10 or fewer streamlines, as in our prior work. Overall 167 of 490 sessions were excluded due to low data quality.<br><br>SVBP:<br>For the SVBP data we implemented a different set of quality assurance steps, as this smaller data set allowed for manual visual inspections of all data. First, consistent with prior work, we quantified the number of outlier slices replaced by FSL's eddy and excluded all sessions with more than 5% outlier slices. Next, we visually inspected all dMRI and R1 data for quality, which led to the removal of six additional sessions due to conspicuous image artifacts or alignment errors. We also visually inspected all bundles of all individuals to confirm that they adhere to their expected anatomical trajectory. No sessions were excluded based on bundle quality issues. Overall, 6 of the 27 sessions were excluded due to data quality concerns.
Non-participation	For the dHCP data we do not have any information about dropout or declined participation, for the locally connected SVBP data, no participant refused to participate during the duration of data collection.
Randomization	In the dHCP dataset, we used partially overlapping cross-sectional and longitudinal samples as described in detail above.<br>For figure 4c, we divided the dHCP data by gender to control for gender effects. For the longitudinal analyses (figure 5) fullterm subjects were matched 1:1 to premature infants according to age and gender.

Reporting for specific materials, systems and methods

We require information from authors about some types of materials, experimental systems and methods used in many studies. Here, indicate whether each material, system or method listed is relevant to your study. If you are not sure if a list item applies to your research, read the appropriate section before selecting a response.

Materials & experimental systems

n/a	Involved in the study
☒	☐ Antibodies
☒	☐ Eukaryotic cell lines
☒	☐ Palaeontology and archaeology
☒	☐ Animals and other organisms
☒	☐ Clinical data
☒	☐ Dual use research of concern
☒	☐ Plants

Methods

n/a	Involved in the study
☒	☐ ChIP-seq
☒	☐ Flow cytometry
☐	☒ MRI-based neuroimaging

Plants

Seed stocks	*Report on the source of all seed stocks or other plant material used. If applicable, state the seed stock centre and catalogue number. If plant specimens were collected from the field, describe the collection location, date and sampling procedures.*
Novel plant genotypes	*Describe the methods by which all novel plant genotypes were produced. This includes those generated by transgenic approaches, gene editing, chemical/radiation-based mutagenesis and hybridization. For transgenic lines, describe the transformation method, the number of independent lines analyzed and the generation upon which experiments were performed. For gene-edited lines, describe the editor used, the endogenous sequence targeted for editing, the targeting guide RNA sequence (if applicable) and how the editor was applied.*
Authentication	*Describe any authentication procedures for each seed stock used or novel genotype generated. Describe any experiments used to assess the effect of a mutation and, where applicable, how potential secondary effects (e.g. second site T-DNA insertions, mosiacism, off-target gene editing) were examined.*

Magnetic resonance imaging

Experimental design

Design type	We used anatomical data only. Infants imaging data was aquired during natural sleep.
Design specifications	n/a
Behavioral performance measures	The Bayley Scales of Infant and Toddler Development, 3rd Edition (Bayley-III) from 215 subjects in the dHCP dataset, at 17-25 months of age.

Acquisition

Imaging type(s)	diffusion and structural MRI
Field strength	3T

Sequence & imaging parameters

dHCP:
Anatomical MRI: Imaging parameters were optimized for contrast to noise ratio using a Cramer Rao Lower bound approach with nominal relaxation parameter values for gray matter T1/T2: 1800/150 ms and white matter T1/T2: 2500/250 ms. T2w and inversion recovery T1w multi-slice FSE images were each acquired in sagittal and axial slice stacks with in-plane resolution 0.8 × 0.8 mm2 and 1.6 mm slices overlapped by 0.8 mm (except in T1w Sagittal which used a slice overlap of 0.74 mm). Other parameters were–T2w: TR/TE = 12000/156 ms, SENSE factor 2.11 (axial) and 2.60 (sagittal); T1w: TR/TI/TE = 4795/1740/8.7 ms, SENSE factor 2.27 (axial) and 2.66 (sagittal). 3D MPRAGE images were acquired with 0.8 mm isotropic resolution and parameters: TR/TI/TE = 11/1400/4.6 ms, SENSE factor 1.2 RL (Right-Left).

Diffusion MRI: The dMRI acquisition was optimized for the properties of the developing brain (Tournier et al., 2020) and implemented as a uniformly distributed set of directions on 4 shells (b = 0 s/mm2: 20, b = 400 s/mm2: 64, b = 1000 s/mm2: 88, b = 2600 s/mm2: 128), each of which was split into 4 optimal subsets acquired using AP, PA, RL, and LR phase encoding. The diffusion gradient b-values and directions and the phase encoding directions were spread temporally taking the risk of infant motion and gradient duty cycle considerations into account in order to achieve maximal imaging efficiency. If the subject woke up during the diffusion scan, the acquisition could be halted and restarted (after resettling the subject) with a user defined overlap in acquired diffusion weightings. The EPI sequence uses MB factor 4, SENSE factor 1.2, partial Fourier factor 0.86, in-plane resolution 1.5 × 1.5 mm, 3 mm slices with 1.5 mm overlap, TE = 90 ms, TR = 3800 ms. Image reconstruction used a dedicated SENSE algorithm.

SVBP:
Anatomical MRI: T2-weighted images were acquired and used for tissue segmentations. T2-weighed image acquisition parameters: TE = 124 ms; TR = 3650 ms; echo train length = 120; voxel size = 0.8 mm3; FOV = 20.5 cm; Scan time: 4 min and 5 s.

Quantitative MRI: An inversion-recovery EPI (IR-EPI) sequence was used to estimate relaxation time (R1) at each voxel. Spoiled-gradient echo images (SPGRs) were used together with the EPI sequence to generate whole-brain synthetic T1-weighted images. We acquired 4 SPGRs whole-brain images with different flip angles: α = 4°, 10°, 15°, 20°; TE = 3 ms; TR = 14 ms; voxel size = 1 mm3; number of slices = 120; FOV = 22.4 cm; Scan time: 4 times ~5 min. We also acquired multiple inversion times (TI) in the IR-EPI using a slice-shuffling technique: 20 TIs with the first TI = 50 ms and TI interval = 150 ms as well as a second IR-EPI with reverse-phase encoding direction. Other acquisition parameters were: voxel size = 2 mm3; number of slices = 60; FOV = 20 cm; in-plane/through-plane acceleration = 1/3; Scan time = two times 1:45 min.

Diffusion MRI: The dMRI data was obtained with the following parameters: multi-shell, #diffusion directions/b-value = 9/0, 30/700, 64/2000; TE = 75.7 ms; TR = 2800 ms; voxel size = 2 mm3; number of slices = 60; FOV = 20 cm; in-plane/through-plane acceleration = 1/3; scan time: 5:08 min. A short dMRI scan was obtained with reverse phase encoding direction and only 6 b=0 images (scan time 0:20 min).

Area of acquisition	Whole brain

Diffusion MRI ☒ Used ☐ Not used

Parameters

dHCP: The dMRI acquisition was optimized for the properties of the developing brain and implemented as a uniformly distributed set of directions on 4 shells (b = 0 s/mm2: 20, b = 400 s/mm2: 64, b = 1000 s/mm2: 88, b = 2600 s/mm2: 128), each of which was split into 4 optimal subsets acquired using AP, PA, RL, and LR phase encoding (Hutter et al., 2018). The EPI sequence uses MB factor 4, SENSE factor 1.2, partial Fourier factor 0.86, in-plane resolution 1.5 × 1.5 mm, 3 mm slices with 1.5 mm overlap, TE = 90 ms, TR = 3800 ms.

SVBP: dMRI data was obtained with the following parameters: multi-shell, #diffusion directions/b-value=9/0, 30/700, 64/2000; TE=75.7ms; TR=2800ms; voxel size=2mm3; number of slices=60; FOV=20cm; in-plane/through-plane acceleration=1/3; scan time: 5:08min. We also acquired a short dMRI scan with reverse phase encoding direction and only 6 b=0 images (scan time 0:20min).

Preprocessing

Preprocessing software

dHCP: Here we used data that was already preprocessed by the dHCP (for more information see Edwards et al., 2022). From there on diffusion MRI tractography was conducted using MRtrix and an automated fiber quantification tool specifically designed to identify fiber bundles in the infant brain (pyBabyAFQ). Cloudknot software was used to deploy both tractography and bundle identification to the Amazon Web Services (AWS) Batch service. Mapping the endpoints of each streamline of each bundle at the gray-white matter interface, was done by using the tckmap function provided by MRtrix. Endpoint density maps were projected onto the cortical surface using FreeSurfer.

SVBP: T1- and T2-weighted images for each participant were rigidly aligned and tissue segmentation into gray and white matter was performed with iBEAT V2.0, followed by manual correction of white matter masks in ITKgray. The edited segmentations were then used to reconstruct cortical surfaces with Infant FreeSurfer. Diffusion MRI data were preprocessed in MRtrix3 (https://github.com/MRtrix3/mrtrix3). Data were denoised using principal component analysis, and susceptibility distortions were corrected with FSL's topup using a reversed phase-encoded image. Motion and eddy-current artifacts were corrected with FSL's eddy, which also detected and replaced outlier slices. Bias field correction was applied using ANTs (https://picsl.upenn.edu/software/ants/). The resulting diffusion data were rigidly registered to the T2-weighted anatomical image, and all alignments were visually inspected for accuracy.
IR-EPI data were used to estimate R1 (R1=1/T1) in each voxel. First, susceptibility-induced distortions were corrected with FSL's top-up and the IR-EPI acquisition with reverse-phase encoding direction. The distortion corrected images were then used to fit the T1 relaxation signal model using a multi-dimensional Levenberg-Marquardt algorithm. From the T1 estimate, we calculated R1 (R1=1/T1) at each voxel. R1 was projected onto the cortical surface using volume-to-surface mapping in FreeSurfer.

Normalization

No normalization; data were analyzed in each individual's native brain space at each time point

Normalization template

Data were not normalized

Noise and artifact removal

Motion and eddy-current artifacts were corrected with FSL's eddy, which also detected and replaced outlier slices.

Volume censoring

n/a

Statistical modeling & inference

Model type and settings

We related myelin-sensitive imaging metrics along white matter bundles and their corresponding cortical targets. First, we examined the relationship between mean T1w/T2w and mean R1 in white and gray matter across bundles using the Pearson's correlation coefficient (r). To determine a chance-level for these correlations, we also computed correlations across tissues in 1,000 iterations of shuffled bundle–target pairings (omitting the true pairings and the homologous bundle in the other hemisphere) and then evaluated if the observed true correlations fell outside the 95% confidence intervals of these random correlations. To illustrate inter-individual variability, we also performed subject-wise Pearson correlations and identified two representative cases showing low and high correlation values in each metric. Additionally, we examined the relationship between T1w/T2w and R1 across bundles in white and gray matter, using the Pearson's correlation coefficient (r).
To further characterize developmental trajectories, we computed T1w/T2w and R1 developmental slopes for each bundle and their cortical terminations by relating these metrics to gestational age at scan (in weeks) (models: T1w/T2w ~ 1 + scan age, R1 ~ 1 + scan age). Paired t-tests were conducted to assess differences in slopes between tissue types. Pearson correlations were employed to test for a relationship between developmental slopes of white matter bundles and their respective cortical targets. To determine a chance-level for these correlations, we also computed correlations across tissues in 1,000 iterations of shuffled bundle–target pairings (omitting the true pairings and the homologous bundle in the other hemisphere) and then evaluated if the observed true correlations fell outside the 95% confidence intervals of these random correlations. We assessed the relationship between the slopes of T1w/T2w and the slopes of R1 development across bundles in white and gray matter using the Pearson's correlation coefficient (r). To ensure that slopes were fit across the same age range, we only included those subjects of the dHCP (N=183) that fell within the (narrower) age range of the SVBP.
As we found large inter-individual differences in the correlation of T1w/T2w across tissues, we aimed to further characterize these inter-individual variability in the large-scale dHCP data. For this we related the strengths of each individual subject's T1w/T2w coupling, i.e. the correlation in T1w/T2w across tissues, to i) gestational age at birth, ii) gestational age at scan, and iii) sex. Next we tested if these inter-individual differences have behavioral consequences. For this, we used a subsample of infants which also completed the Bayley Scales of Infant and Toddler Development, Third Edition (Bayley-III). We examined three behavioral domains (cognition, language and motor) by taking the sum of age-standardized scores within each respective Bayley-III subscale and related these behavioral outcomes to the correlation of T1w/T2w across white and gray matter at birth. In addition, we also tested if T1w/T2w measured in each tissue separately relates to later behavioral outcomes. For each measure, Pearson correlations were used to assess associations, and the coefficient of determination (r^2) was reported for each model. Bonferroni correction was applied. Finally, as we found a significant correlation between T1w/

T2w coupling across tissues and motor outcomes, we also assess the contribution of demographic variables on this relationship. To this end, we performed a series of nested linear model comparisons. A baseline model predicting standardized Bayley-III motor scores from the T1w/T2w coupling was contrasted against three extended models that each included one additional covariate: scan age, birth age, or sex. All models were fit using ordinary least squares regression implemented in Python (statsmodels package). Model comparisons were evaluated using ANOVA-based F-tests, testing whether inclusion of each covariate significantly improved model fit relative to the baseline model. Statistical significance was set at $p < 0.05$ (two-tailed).

To examine the impact of preterm birth and postnatal experience on the correlation of T1w/T2w across tissues, we compared three groups in a small longitudinal sub-sample: (i) preterm infants scanned shortly after birth, (ii) the same preterm infants scanned again at term-aquivalent age, and (iii) full-term infants individually matched to the preterm infants second scan on sex and scan age. For each group, we computed the mean T1w/T2w values in white matter bundles and corresponding cortical targets, and assessed their linear relationship using Pearson's correlation. To test if the correlation of T1w/T2w across tissues differs between the groups we employed a nonparametric bootstrap procedure. For each group, we generated 1,000 bootstrap samples by drawing, with replacement, $n = 26$ observations and then computed the correlation of T1w/T2w across tissues in each sample. To compare correlations across the three groups, we examined the nonparametric bootstrap distribution of their pairwise differences in r^2, assessing whether zero lay within or outside the 95% confidence intervals. When 0 fell outside the confidence intervals, this was interpreted as a significant group difference.

Effect(s) tested

Relationship between mean T1w/T2w and R1 in white and gray matter across bundles (Pearson's correlation coefficient)
Subject-wise Pearson correlations, identifying two representative cases showing low and high correlation values
Relationship between behavioral performance (motor, cognition, language) and the correlation between T1w/T2w in gray and white matter
Correlation of the slopes of T1w/T2w and R1 growth along white matter bundles and their gray matter targets
Relationship between T1w/T2w of white matter bundles and their respective gray matter targets in three groups: a) infants born preterm scanned at birth; b) infants born preterm scanned at ≥ 37 weeks gestational age; c) age-matched infants born full-term
Bootstrap distributions of correlation differences between the groups above

Specify type of analysis: ☐ Whole brain ☒ ROI-based ☐ Both

Anatomical location(s): White matter bundles and their endpoints in cortex

Statistic type for inference (See Eklund et al. 2016)

Statistical test were performed at the level of entire bundles and their cortical terminations

Correction

Bonferroni correction was applied where applicable

Models & analysis

n/a	Involved in the study
☒	☐ Functional and/or effective connectivity
☒	☐ Graph analysis
☒	☐ Multivariate modeling or predictive analysis
