# Supplementary material for: Cortical and white matter myelination proceed in concert during early infancy
Source: Nat Commun. 2026 Jun 17;17:5353. doi: 10.1038/s41467-026-73366-9 (PMC13276173; doi:10.1038/s41467-026-73366-9)
Supplement: Supplementary file 3 — Transparent Peer Review file [file 41467_2026_73366_MOESM3_ESM.pdf]

nature portfolio

Peer Review File

Cortical and white matter myelination proceed in concert during early infancy

Corresponding Author: Ms Stephanie Zika

This file contains all reviewer reports in order by version, followed by all author rebuttals in order by version.

Version 0:

Reviewer comments:

Reviewer #1

(Remarks to the Author)
The study by Zika et al. provides an interesting and valuable investigation into the developmental relationships between gray matter (GM) and white matter (WM) T1w/T2w ratios, interpreted here as a proxy for myelin content. The authors demonstrate that mean T1w/T2w values across GM and WM, as well as their trajectories, are coupled in early development, and suggest that prematurity impacts these relationships with possible links to later outcomes.
The work has several clear strengths: a large sample size, careful bundle extraction, quantification of T1w/T2w values in GM and WM, and inclusion of both cross-sectional and longitudinal cohorts. The introduction is well written and demonstrates a thorough background search. However, I find the discussion and methods bit too lengthy. More importantly, I have concerns regarding aspects of the analysis and interpretation, which I detail below:

Major comments:
Interpretation of T1w/T2w, introduction, and framing
1. The authors acknowledge that T1w/T2w as a measure of myelin even in the WM is debated, this applies even more strongly to cortex. It would be better to use more cautious language in the introduction when presenting T1w/T2w as an indicator of myelin, given that the entire study builds on this interpretation.
2. The discussion should also more explicitly acknowledge that correlated developmental trajectories of GM and WM T1w/T2w could reflect coinciding maturational changes beyond myelination (this is indicated in studies cited in the discussion which correlation diverse cortical metrics with WM development). While the observations are not inconsistent with activity-dependent myelination, this remains speculative.
3. I really appreciate the detailed overview of myelination trajectories in the introduction, and generally thoroughness of the introduction. However, the last two paragraphs read more like a results/discussion section than a statement of aims or hypotheses. For example, “We propose that the observed link between gray and white matter myelination is most readily accounted for by the theory of activity-dependent myelination” is difficult to accept prior to results. Moving this material to the discussion would free space to give a quick introduction of overall goals, overview of methods and cohort details earlier (rather than in results in case of cohorts, or only at the very end in methods), Incidentally, I think the supplementary figure 3S would be very useful in the main body.
4. I encourage more discussion of bundle-specific rates of T1w/T2w change across the studied period. This could highlight clusters of bundles undergoing rapid maturation and situate findings in the context of known developmental sequences (which were presented in the introduction).
Methods clarity and quality control
5. How was quality of bundle identification assessed, especially in very preterm scans? Was quality related to age at scan, and could measurement noise vary across the relatively wide scan-age range? For tissue response function estimation: WM/GM components are strongly influenced by PMA at scan. It would be helpful to elaborate (in the methods, or here because I am curious) on how response functions were derived and whether age-related differences impacted tractography, especially in younger subjects. Were smaller or more complex bundles disproportionately affected?
6. Were cortical/subcortical delineations used for bundle endpoints checked against the surface parcellations? Would mismatches impact bundle–cortex coupling measures?
7. I am sorry, but upon reading it’s hard to understand how bundle-wise GM/WM correlation within subject was computed. I thought you have one measure for GM and for WM per bundle per subject?
8. Wondering if there was any correction for multiple comparisons throughout the paper, I assume yes but it would be good to state explicitly what was corrected for what when you are presenting the results.

Prematurity and timing effects
9. Was prematurity related to time between birth and scan? (for very early preterms it might be difficult to scan close to birth). Would this interact with your observations?
10. I am struggling to interpret the results in the section “T1w/T2w coupling across tissues is linked to infants’ motor skills later in life” and the related conclusion (iv). The analyses appear to rely on a cross-sectional sample spanning a wide developmental age range. For this period, both age at scan and GA at birth should be carefully controlled when relating imaging measures to later outcomes (and age at assessment as well, if raw Bayley scores were used, as mentioned in the methods). This is especially important given your other conclusion that “In measurements conducted shortly after birth, coupling between T1w/T2w in white and gray matter is weaker at preterm than at full-term birth”. In other words, what may appear as a coupling–outcome association could instead reflect the effect of prematurity: infants scanned earlier (more premature) both show lower scores and weaker coupling, without coupling itself being the factor. To support the claim that lower coupling per se predicts lower motor outcomes, the analyses should at minimum adjust for age at scan (and ideally GA at birth). Even better, you could restrict the analysis to infants scanned at term (>37 wGA) to reduce this confound. I would also strongly recommend plotting GA at birth and/or scan against Bayley scores to clarify this relationship. As currently framed, the conclusion that “higher coupling predicts better motor skills” seems insufficiently justified without these adjustments.
11. ‘This suggests that the link between T1w/T2w across tissues is dependent on brain maturity as well as on experience. After all, at birth, the brains of preterm infants had less time to mature compared to their full-term born peers, which could explain the observed reduced T1w/T2w coupling. Indeed, research indicates that preterm birth can alter both the speed and degree of brain myelination, potentially leading to developmental differences when compared to full-term infants’ This is strangely worded. Reduced coupling shortly after preterm birth compared to full-term could simply reflect typical maturational differences, as 34wGA brains are less mature than 40wGA. Comparing preterm infants at TEA to full-term neonates is the true test of prematurity effects.
12. The discussion of sensory experience in preterm infants could benefit from reframing. Rather than simply receiving “more” input, these infants experience qualitatively different, often noxious, sensory stimulation, which may dysregulate development (in contrast to typical in-utero development which has plenty of sensory experience, which is necessary for optimal maturation). Stronger T1w/T2w coupling does not necessarily indicate “better” outcomes (this is also important in context of ‘seemingly surprising relationship between coupling and later outcomes versus prematurity’ that you mention in the discussion) especially without taking into account the wide age range in the cross-sectional sample which I suggested above might be interacting with the outcome results.
Analyses and figures
13. Figure 2: please indicate ages at scan for subjects B, C, D. Could apparent correlations reflect age or bundle quality?
14. Correlation coefficients are sensitive to variance: bundles with narrow T1w/T2w ranges will give lower r. It is difficult to compare the results across bundles without visualizing distributions. A boxplot or similar visualization of raw T1w/T2w values across bundles would be very helpful. In the same vein, also during typical development, if we assume there are no methodological artefacts related to GA, one might expect lower individual variability that increases with age, would this play a role in estimation of ‘lower coupling’ at earlier ages?
15. Figure 5: does the difference between panels B and C largely reflect CCL/CCR? Is there any reason why you think CC diverges so much between the two groups?
16. Bayley-III: Please justify using raw score sums rather than age-corrected composite or scaled scores. Raw scores are not age-adjusted and could confound developmental interpretations to my knowledge?
Minor comments
• ‘(GA at birth: mean SD: 32.04 ± 2.97 weeks)’ -> ‘(GA at birth: mean ± SD: 32.04 ± 2.97 weeks)’
• ‘GA of at least 37 weeks (GA at second scan…)’ you refer to this groups as ‘preterm infants scanned at term-equivalent age’ later on, it would be helpful to homogenise across the manuscript
• “Overall, these results suggest that white and gray matter are developing in concert during early infancy. .” → remove extra period.
• Matter of personal taste, Figure 3: consider omitting regression lines for nonsignificant relationships as this might be confusing at a glance. Showing thresholds of atypicality for Bayley scores would contextualize results, since most toddlers within dHCP perform within the typical range (although I am not sure this is possible with raw scores).
• It might make figures more readable if you used same colour for bilateral bundles but used a different shape for left, and right. This would significantly simplify the colour scheme making it easier to identify bundles on scatter plots.
• Discussion: reads well, but should be tightened for clarity and focus (see analysis-related comments). Similarly, the data section can be shortened, no need for extensive background on dHCP beyond what data were used.

I hope my comments are helpful in refining the manuscript and clarifying the analyses.
I am looking forward your responses,
Andrea Gondova

(Remarks on code availability)
The authors have provided their code and Jupyter notebooks, including links to pipelines for tractography, bundle identification, and cortical endpoint mapping, which will be very helpful for others to reproduce and build upon these analyses. (For transparency, I selected ‘No’ for “reviewed the code” because, while I checked that all the described sections were present in the GitHub folder, I did not review the code in detail.)

Reviewer #2

(Remarks to the Author)

This study examines relationships between white and cortical gray matter T1w/T2w in a large cohort of infants from the Developing Human Connectome. In particular, the authors investigated the degree to which the white and cortical gray matter T1w/T2w are inter-related across a number of white matter bundles, how these are shaped by early development, and if the T1w/T2w correlation/coupling is related to later infant behavior/cognition. The authors observe both the mean and rates of changes of the T1w/T2w of white matter bundles to be correlated with the T1w/T2w of the cortical "targets" and the correlation between T1w/T2w white and gray matter associated with improved motor skills later in childhood. The authors also compare these relationships/patterns between preterm, preterm at term-equivalent-age (TEA) and full-term infants, observing the correlations to be higher in the TEA infants than the full-term. The authors argue that these patterns highlight that the changes in T1w/T2w in early infancy are strongly white and gray matter. Overall, the authors perform an interesting analysis on a large infant sample. However, I have several questions around the method and analyses performed that should be clarified. The paper is also quite dense and long and I think there are areas where the text should be clarified and/or reduced.

Comments:

1) While the dHCP pipelines were used for the preprocessing of data, it would be useful/informative to include additional information about these steps. For example, was the bias field correction performed separately on the T1w and T2w images? How do these steps effect the T1w/T2w ratios? Was the registration between the T1w and T2w images a rigid-body registration or were more degrees of freedom used?

2) How are intensity differences between subjects controlled for or handled with the T1w/T2w ratio. Specifically, given that different scanner settings will influence the individual intensities of a given T1w or T2w image (e.g. primary reason why one cannot compare T1w or T2w intensities across subjects), its not clear how just the ratio between T1w and T2w images accounts for such differences. For example, Gazetti et al., (Ganzetti et al., 2014) describe an 'intensity standardization' procedure after bias correction for computing T1w/T2w ratios to help standardize values across subjects. Is a similar standardization performed here or how

3) The authors should be careful to refer to the T1w/T2w ratio as a measure of myelination. As they acknowledge, no MR measure directly measures myelin and other quantitative imaging techniques, like relaxometry/myelin water fraction imaging, or quantitative magnetization transfer imaging methods, appear more sensitive to myelin content than the T1w/T2w ratio (see Uddin et al., 2018; 2019). Moreover, while R1 is more sensitive to myelin, the statement that because R1 is higher in the cortex in infants there is more myelin content in the cortex seems inaccurate. When examining other studies using myelin water imaging or magnetization transfer techniques, this is not the case. Moreover, while R1/qT1 is sensitive to myelin, there are a number of other things that directly influence R1 (cytoarchitecture, water content, etc…).

4) What was the purpose for performing Tractography when the pyBabyAFQ is being used to generate the white matter bundles? This was unclear in how this is being used. Was it for the cortical terminations? If so, was it ensured that the cortical terminations were consistent with the bundles from the pyBabyAFQ?

5) Presumably the diffusion MRI data need to be registered to the anatomical data to be able to extract the T1w/T2w from the bundles. Was this performed as part of the dHCP pipelines or how was this done?

6) I would suggest creating a figure that illustrates the process for generating the white matter and cortical T1w/T2w measures.

7) In the discussion of the limitations, the authors state: "It is important to recognize, however, that the myelin content of infant white matter more closely resembles that of adult cortex than adult white matter, which has been shown by comparing R1 values across tissues from different age groups." I disagree with this sentence in comparing the infant white matter to the adult cortex and using this as justification. I also don't understand how this statement helps support the use of T1w/T2w in infants.

(Remarks on code availability)
I reviewed the available GitHub repositories and the code there seemed appropriate. I did not try to install or run the code, however.

Reviewer #3

(Remarks to the Author)
This interesting work looks at how a measure of tissue structure, the T1w/T2w ratio, changes concurrently with developmental age in both the white matter and the associated grey matter. It uses a set of known white matter bundles and their cortical targets to do this. It looks at the developmental trajectories of these associations and compares individual variability in how related these measures are within-subject to relate to outcomes at 18 months, and how they differ in prematurity.

It's a really nice way of looking at regional brain development but I've a few large concerns on how the data has been interpreted and how specific the age associations are.

Biology - There is a focus here on T1/T2 reflecting myelination. Even in adults, this value is criticised as not mapping to

other (more validated) measures of myelination, especially in white matter (referenced by the authors here). In neonatal white matter in particular, outside of the primary sensory regions, the majority of signal change is more likely to be a reduction in water content as opposed to an increase in myelination. This is quite critical as the paper is very focused on testing a hypothesis of activity dependent myelination but I don't think this can be tested without one of the more specific white matter imaging techniques which can separate out the larger water signal. An example of a paper from this group (ref 59) using T1w/T2w in the same cohort showed that it is useable in this age group but not that it's specific to myelin.

Specificity to regional vs global development - are the associations particularly different (across subjects) if you pick a different cortical target ROI for a fixed white matter bundle? i.e. Is it just individual differences in global T1 and T2w ratio as opposed to anything specific to the bundle?

Use of raw Bayley scores - why were raw scores chosen? It seems like an unnecessary extra confound to add as these scores will scale on average with age of assessment. Did this test include age at scan / sex? Also were there sex differences?

The corticospinal tract - this tract is a bit of an outlier to the others, in that it has substantially higher values in both GM and WM which does reflect it's higher myelin value. What happens to these associations when it is not included (e.g. figure 4).

(Remarks on code availability)
I have looked at the code, set up as python notebooks. I don't know the syntax etc to the AFQ programmes but, in general, looks good. There isn't much guidance in the notebooks but would be easy enough to add.

Version 1:

Reviewer comments:

Reviewer #1

(Remarks to the Author)
I would like to thank the authors for their thorough responses and for all the additional analyses performed in response to the earlier comments – they fully answered my concerns. I especially appreciate the inclusion of the parallel quantitative R1 dataset. It is reassuring to see that similar GM/WM coupling patterns emerge across both measures.

Few very minor comments which I think can be taken care of at editing stage:
'We find that white matter and cortical myelin contents are tightly coupled: For both R1 and T1w/T2w values within each' : or f?
'Further, in a longitudinal sub-sample, we find lower correlation of T1w/T2w across gray and white matter in preterm compared to full-term infants at birth..' one dot too many.
'tissue response functions were computed': averaged over all subjects in the study? For the oldest subjects? Or you used individual responses?
'Are you perhaps referring to figure 3a-b?' Thank you, your clarification helps but I was actually referring to Fig 2. where you introduce: 'Mean correlation with T1w/T2w values averaged across subjects' I though somehow the correlation was meaned. To confirm, a,b are: Correlation of mean T1w/T2w bundle values averaged across subjects?
'If gestational age was the main predictor of motor skill, we would have expected that adding this factor would improve the model'. I would caution against this interpretation. If T1w/T2w coupling and GA are strongly correlated (as your results indicate), then adding GA to a model that already includes coupling would not necessarily improve model fit, even if GA were an important predictor. This simply reflects the fact that the second predictor contributes little unique variance when two variables share substantial variance. To assess whether coupling explains variability in motor outcomes beyond what can be attributed to GA, it may be more informative to directly compare models predicting motor outcome from coupling alone versus GA alone, or to examine partial correlations (outcome ~ coupling, controlling for GA).I am not disagreeing with the revised, more cautious conclusion -> this seems appropriate. I am including this reflection primarily for future reference regarding the modelling approach.

(Remarks on code availability)
I have checked the repository and skimmed through the code, but did not try to run it.

Reviewer #2

(Remarks to the Author)
I thank the authors for their corrections and clarifications to the previous review. I believe the changes that the authors have been made have substantially improved the manuscript. The addition of the local sample, including quantitative R1 and analyses between T1w/T2w and qR1, is informative. The manuscript and additions are well described. I have no further comments.

(Remarks on code availability)

I briefly reviewed the code in the available repo. The code looks to be well documented and represents the work that was performed. I did not try to install/run the code.

Open Access This Peer Review File is licensed under a Creative Commons Attribution 4.0 International License, which permits use, sharing, adaptation, distribution and reproduction in any medium or format, as long as you give appropriate credit to the original author(s) and the source, provide a link to the Creative Commons license, and indicate if changes were made.
In cases where reviewers are anonymous, credit should be given to 'Anonymous Referee' and the source.
The images or other third party material in this Peer Review File are included in the article's Creative Commons license, unless indicated otherwise in a credit line to the material. If material is not included in the article's Creative Commons license and your intended use is not permitted by statutory regulation or exceeds the permitted use, you will need to obtain permission directly from the copyright holder.
To view a copy of this license, visit https://creativecommons.org/licenses/by/4.0/

Reviewer #1:

The study by Zika et al. provides an interesting and valuable investigation into the developmental relationships between gray matter (GM) and white matter (WM) T1w/T2w ratios, interpreted here as a proxy for myelin content. The authors demonstrate that mean T1w/T2w values across GM and WM, as well as their trajectories, are coupled in early development, and suggest that prematurity impacts these relationships with possible links to later outcomes.
The work has several clear strengths: a large sample size, careful bundle extraction, quantification of T1w/T2w values in GM and WM, and inclusion of both cross-sectional and longitudinal cohorts. The introduction is well written and demonstrates a thorough background search. However, I find the discussion and methods bit too lengthy. More importantly, I have concerns regarding aspects of the analysis and interpretation, which I detail below:

Thank you for your detailed and helpful evaluation. Based on your feedback we have shortened the discussion and reworked the method section for brevity and clarity, while also ensuring that all methodological details are adequately presented. We also added new analyses and a more cautious interpretation of the findings as detailed below in response to each of your specific comments. We feel that overall these changes have substantially strengthened the manuscript.

Major comments:
Interpretation of T1w/T2w, introduction, and framing
1. The authors acknowledge that T1w/T2w as a measure of myelin even in the WM is debated, this applies even more strongly to cortex. It would be better to use more cautious language in the introduction when presenting T1w/T2w as an indicator of myelin, given that the entire study builds on this interpretation.

Thank you for this important comment. We agree that the use of T1w/T2w as a proxy for myelin requires further validation. As such, we have now added a complementary quantitative neuroscience dataset that, in addition to dMRI also includes qMRI data, and hence enabled analyses of R1. Quantitative R1 is widely accepted as a proxy for myelin and its direct relationship with myelin content has been confirmed in post-mortem data (see e.g. Schüber el al., Neuroimage, 2014). Using these complementary data we found striking consensus between T1w/T2w and quantitative R1. Most importantly, as shown in new figure 2 and 3 included below, both T1w/T2w and R1 show correlated mean values and developmental slopes across tissues. The newly added R1 analyses hence further supports our claim of coupled myelin development across tissues with a more widely accepted and validated measure. These new results are presented in detail in the first two paragraphs of the results sections.

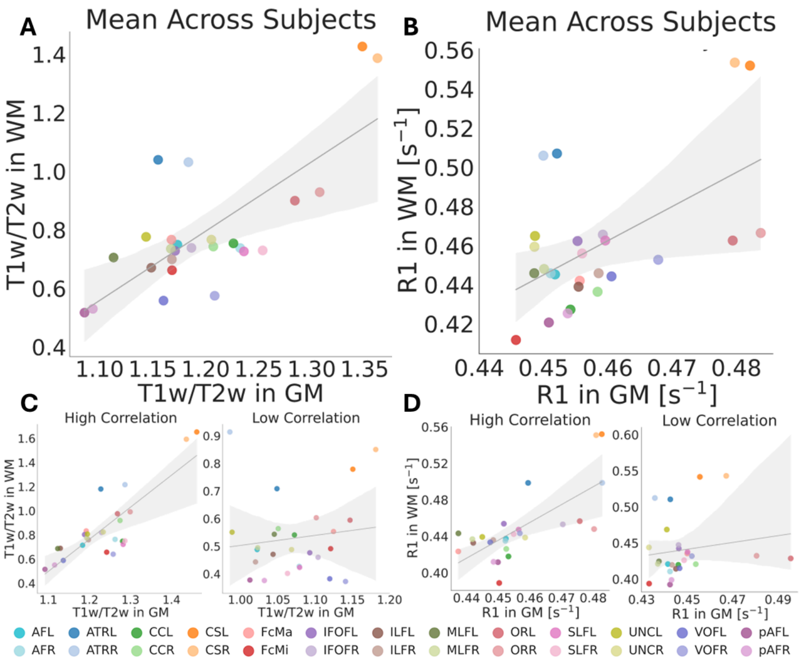

Figure 2. **Relationship between T1w/T2w and R1 of WM bundles and their respective GM targets.** Each dot is a bundle. **a** Mean correlation with T1w/T2w values averaged across subjects (r^2=0.55, p-value=1.45e^{-5}). **b** Mean correlation with R1 values averaged across subjects (r^2=0.32, p-value=0.002). **c** Example individual subjects exhibiting high (left; GA at scan: 41.43 weeks; r^2=0.68, p-value=2.28e^{-7}) and low (right; GA at scan: 31.43 weeks; r^2=0.017, p-value=0.52) correlation between T1w/T2w of white and gray matter. **d** Example individual subjects exhibiting high (left; GA at scan: 42.5 weeks; r^2=0.51, p-value=4.21e^{-5}) and low (right; GA at scan: 40.4 weeks; r^2=0.03, p-value=0.44) correlation between R1 of white and gray matter. Abbreviations: WM: white matter, GM: gray matter, AF: Arcuate Fasciculus, ATR: Anterior Thalamic Radiation, CC: Cingulum Cingulate, CS: Cortico-Spinal Tract, FcMa: Forceps Major, FcMi: Forceps Minor, IFOF: Inferior Frontal Occipital Fasciculus, ILF: Inferior Longitudinal Fasciculus, MLF: Middle Longitudinal Fasciculus, OR: Optic Radiation, SLF: Superior Longitudinal Fasciculus, UNC: Uncinate Fasciculus, VOF: Ventral Occipital Fasciculus, pAF: Posterior Arcuate Fasciculus, L=left, R=right.

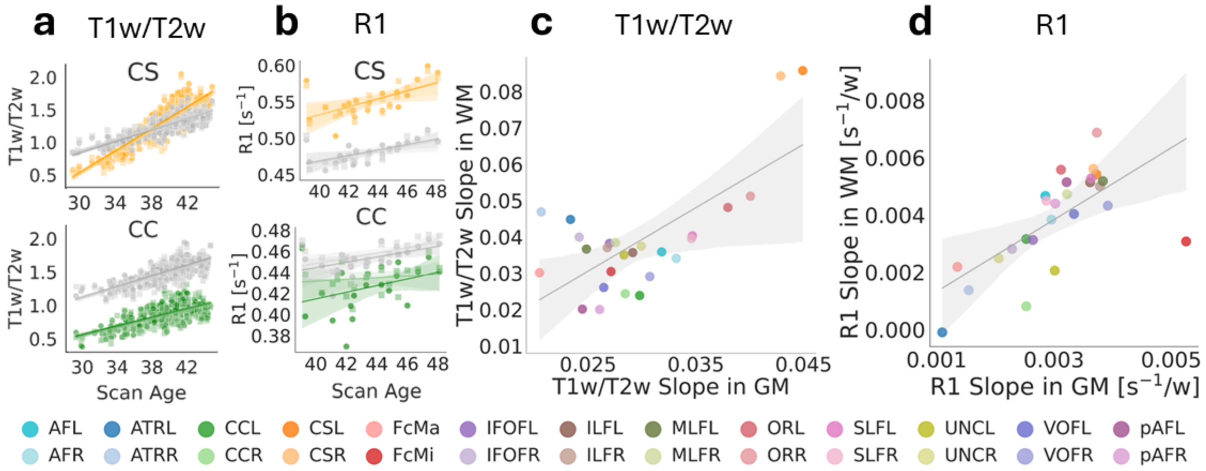

Figure 3: **The rates of change of myelin-sensitive imaging metrics are correlated across white and gray matter. a,b** Two example bundles (cortico-spinal tract (CS, top) and cingulum cingulate (CC, bottom)) illustrate T1w/T2w (a) and R1 (b) slopes in gray and white matter (for all bundles see Supplementary Figures S6 and S7). Gray circles indicate gray matter values in each subject, colored circles represent white matter values in each subject, and the steepness of the lines indicate the slopes (rates of change relative to the infant's gestational age at the time of measurement in weeks), the two hemispheres are presented in different shades. **c,d** Relationship between the slopes of T1w/t2w (c) and R1 (d) along white matter (WM) bundles and their gray matter (GM) targets (T1w/T2w: r^2=0.49, p-value=6.28e^{-5}; R1: r^2=0.45, p-value=0.0002). Each circle indicates a bundle, the two hemispheres are presented in different shades. Abbreviations: AF: Arcuate Fasciculus, ATR: Anterior Thalamic Radiation, CC: Cingulum Cingulate, CS: Cortico-Spinal Tract, FcMa: Forceps Major, FcMi: Forceps Minor, IFOF: Inferior Frontal

Occipital Fasciculus, ILF: Inferior Longitudinal Fasciculus, MLF: Middle Longitudinal Fasciculus, OR: Optic Radiation, SLF: Superior Longitudinal Fasciculus, UNC: Uncinate Fasciculus, VOF: Ventral Occipital Fasciculus, pAF: Posterior Arcuate Fasciculus, L=left, R=right.

2. The discussion should also more explicitly acknowledge that correlated developmental trajectories of GM and WM T1w/T2w could reflect coinciding maturational changes beyond myelination (this is indicated in studies cited in the discussion which correlation diverse cortical metrics with WM development). While the observations are not inconsistent with activity-dependent myelination, this remains speculative.

Thank you. We agree that the role of activity dependent myelination in driving the correlated development across tissues remains speculative at this point. As such, we have substantially edited the manuscript and largely removed this conclusion. We now mention this only as one of multiple hypotheses in the discussion (on page 11, lines 329-343), where we write:
"Here we find that T1w/T2w and R1 development is tightly linked between white matter bundles and their corresponding cortical targets. We hypothesize that this observation might be accounted for by the theory of activity-dependent myelination[25,75]: According to this theory, active neurons send trophic signals (e.g., glutamatergic release, ATP signaling) along their axons that promote oligodendrocyte differentiation both within the cortex and along white matter projections, such that increased neuronal firing can increase myelination in both compartments[76–79] (for review see[80]). Alternative hypotheses for the observed coupling in myelination include: 1) Coordinated genetic regulation: As similar genetic programs direct the proliferation, migration, and differentiation of oligodendrocyte precursor cells (OPCs) in both cortex and white matter[81,82], these genetic influences might drive the coordinated myelination of white and gray matter. 2) Common metabolic and microenvironmental factors: Both cortical and white matter myelination are energy-demanding processes that depend on a supply of lipids and other metabolic substrates[18]. As such, the local availability of these resources, coupled with support from glial cells like astrocytes could concurrently influence myelination in both tissues[83,84].

"

3. I really appreciate the detailed overview of myelination trajectories in the introduction, and generally thoroughness of the introduction. However, the last two paragraphs read more like a results/discussion section than a statement of aims or hypotheses. For example, "We propose that the observed link between gray and white matter myelination is most readily accounted for by the theory of activity-dependent myelination" is difficult to accept prior to results. Moving this material to the discussion would free space to give a quick introduction of overall goals, overview of methods and cohort details earlier (rather than in results in case of cohorts, or only at the very end in methods), Incidentally, I think the supplementary figure 3S would be very useful in the main body.

Thank you for your feedback. In accordance with your suggestion, we have moved supplementary figure S3 to the main manuscript and included it in the method section as new figure 6. In addition, we streamlined the mentioned paragraphs in the introduction and used the freed up space to provide further methodological details. These sections, on page 2 in lines 83-98, now read as follows:

“In this work we combine these approaches to gain both precision and robustness by evaluating a small-scale (N=21) R1 data set collected locally at Stanford University (Stanford VPNL Baby Project (SVBP) data) as well as a large-scale (N=273) T1w/T2w data set shared openly by the Developing Human Connectome Project (dHCP). In both data sets, we test for a link between gray and white matter myelination in early infancy by relating R1 or T1w/T2w of white matter bundles to R1 or T1w/T2w measured at their corresponding cortical targets. We find that white matter and cortical myelin contents are tightly coupled: For both R1 and T1w/T2w values within each bundle are positively correlated with values at its corresponding cortical targets. Similarly, for both R1 and T1w/T2w, the rate of change during early infancy is coupled between white and gray matter. We also observe large inter-individual variability in the strengths of white and gray matter coupling, which, in the dHCP data, is related to gestational age, and correlates with motor performance later in life. In the dHCP data, the coupling between T1w/T2w of white and gray matter is weaker in preterm than full-term infants, even at term-aquivalent age. By highlighting the tight coupling of gray and white matter myelin growths, our findings provide a comprehensive understanding of early life brain myelination and suggest that white and gray matter myelination should be considered conjointly.”

4. I encourage more discussion of bundle-specific rates of T1w/T2w change across the studied period. This could highlight clusters of bundles undergoing rapid maturation and situate findings in the context of known developmental sequences (which were presented in the introduction).

Thank you. In response to your feedback we added 2 tables to the Supplementary Materials that detail the bundle-specific slopes of both T1w/T2w and R1. These tables are included below. We also briefly discuss the differences in slopes across bundles in the results section (page 6, lines 197-210), where we write: “Similar to the mean values reported above, the slopes of T1w/T2w and R1 change in gray and white matter also varied across the bundles. For T1w/T2w, the CS, for instance, stood out with the highest rate of change in both the white matter bundle and at its cortical target. In contrast, the ATR, pAF, and the FcMi exhibit comparatively low rates of change, with the other bundles falling in between. For R1, the CS did not stand out as clearly, but the FcMi and ATR again exhibit comparatively low rates of change, with the other bundles falling in between. Further, again similar to the mean values, bilateral bundles showed similar gray and white matter slopes between the left and right hemisphere in both T1w/T2w and R1. As the slopes of T1w/T2w and R1 are derived from datasets with different scan age ranges, they are not directly comparable. However, even when we matched the scan age ranges (by including only those subjects from the dHCP that are within the same range as the SVBP, N=183), we find that T1w/T2w and R1 slopes are not significantly correlated with each other (Supplementary Figure S8) in either the gray matter (r^2=0.07, p-value=0.20) or the white matter (r^2=0.06, p-value=0.24).”

T1w/T2w

Bundle	WM p	WM r^2	WM coeff	GM p	GM r^2	GM coeff
AFL	3.26E-42	0.49601121	0.03588858	1.01E-62	0.64396946	0.03183006
AFR	5.88E-42	0.49382503	0.0341056	4.83E-56	0.60128482	0.03317838
ATRL	4.40E-66	0.66370581	4.48E-02	2.18E-38	0.46230977	0.02336754
ATRR	7.29E-67	0.66813018	0.04688158	7.72E-30	0.3786821	0.02065157
CCL	7.79E-40	0.47531305	0.02387939	2.94E-61	0.63502846	0.02978607
CCR	5.44E-32	0.40086905	0.02437258	2.42E-46	0.53004997	0.02844867
CSL	1.24E-88	0.77062602	0.08577072	1.77E-74	0.7083524	0.04492214
CSR	5.20E-84	0.751909	0.08429417	2.71E-67	0.67053776	0.04286874
FcMi	5.04E-48	0.54325362	0.03047732	7.63E-31	0.38914321	0.02712702
FcMa	3.30E-56	0.6024077	0.03014956	3.46E-36	0.4419104	0.02049164
IFOFL	4.83E-69	0.68017531	0.03820163	2.13E-44	0.51432327	0.02698286
IFOFR	3.35E-74	0.70697334	0.03996037	6.77E-42	0.49330271	0.02417439
ILFL	2.92E-62	0.64118653	0.03568442	1.29E-50	0.56288097	0.02914318
ILFR	1.86E-66	0.66583242	0.03703395	1.13E-47	0.54052215	0.02678297
MLFL	2.91E-62	0.64119827	0.03665657	3.90E-50	0.55930199	0.02484381
MLFR	1.44E-68	0.67759523	0.03846351	4.64E-49	0.55119713	0.02753962
ORL	1.59E-66	0.66622128	0.04810809	5.59E-38	0.45857353	0.03797427
ORR	1.15E-65	0.6613197	0.05121672	2.41E-40	0.47981063	0.04007292
SLFL	2.46E-52	0.57543018	0.04031047	6.45E-66	0.66275742	0.03468034
SLFR	1.30E-51	0.57020614	0.03956626	4.15E-64	0.65225524	0.03460147
UNCL	2.67E-71	0.69218966	0.03503019	1.19E-58	0.61852777	0.02833703
UNCR	7.06E-80	0.73388298	0.03747226	1.58E-51	0.56957773	0.02993709
VOFL	1.12E-45	0.52473566	0.02609958	5.36E-34	0.42084628	0.02645496
VOFR	1.13E-48	0.54823631	0.02909089	1.70E-39	0.47228797	0.03073249
pAFL	3.70E-39	0.46926914	0.02012696	1.02E-44	0.51695343	0.0244694
pAFR	3.09E-38	0.46092024	0.02004629	1.14E-44	0.51655634	0.02606878

Table S1: **Relationship between gestational age and T1w/T2w in each white matter bundle (WM) and their gray matter (GM) targets.** The table summarizes the statistical relationships between scan age (gestational age at measurement in weeks) and T1w/T2w in each bundle. For each bundle, the correlation (r^2), the coefficient (slope), and the associated p-value is reported separately for white matter (WM) and gray matter (GM). All bundles show statistically significant correlations (all ps < 0.05), as highlighted in green. Abbreviations: GM: gray matter, WM: white matter, AF: Arcuate Fasciculus, ATR: Anterior Thalamic Radiation], CC: Cingulum Cingulate, CS: Cortico-Spinal Tract, FcMa: Forceps Major, FcMi: Forceps Minor, IFO: Inferior Frontal Occipital Fasciculus, ILF: Inferior Longitudinal Fasciculus, MLF: Middle Longitudinal Fasciculus, OR: Optic Radiation, SLF: Superior Longitudinal Fasciculus, UNC: Uncinate Fasciculus, VOF: Ventral Occipital Fasciculus, pAF: Posterior Arcuate Fasciculus, L=left, R=right.

R1

Bundle	WM p	WM r^2	WM coeff	GM p	GM r^2	GM coeff
AFL	0.02197953	0.24675132	0.00466576	0.00649369	0.32962908	0.00285392
AFR	0.04100023	0.20190967	0.00383947	0.00934692	0.30560913	0.00295793
ATRL	0.94401978	0.00026639	-9.69E-05	0.36799227	0.0428421	0.00109592
ATRR	0.30297424	0.05571395	0.0013761	0.20932102	0.08162375	0.00154812
CCL	0.12518794	0.11927374	0.00315954	0.01885071	0.25756295	0.00252821
CCR	0.54563896	0.01953842	0.00081258	0.01501227	0.27340477	0.00252928
CSL	0.00349122	0.36903122	0.00541797	0.00162504	0.41490455	0.00372508
CSR	0.00060239	0.47006738	0.00561202	0.00269811	0.38482106	0.00367237
FcMi	0.24106061	0.07156173	0.00307622	0.25536165	0.06750817	0.00525478
FcMa	0.10296567	0.13379251	0.00218789	0.00173945	0.41094313	0.00135454
IFOFL	0.0268136	0.23261049	0.00312842	0.04238307	0.19948637	0.00264276
IFOFR	0.01881613	0.25769162	0.00281515	0.03487151	0.21368809	0.00228502
ILFL	0.00148988	0.41992715	0.00514167	0.00211819	0.39934153	0.00361781
ILFR	0.00011579	0.55135401	0.00500508	0.00095596	0.44500272	0.00378354
MLFL	0.00132246	0.42675957	0.00518177	0.00054973	0.47490963	0.00383553
MLFR	0.00077035	0.4568491	0.00472878	0.00243026	0.39113109	0.0032256
ORL	0.00748707	0.32031908	0.00558123	0.10497975	0.13235144	0.00312207
ORR	0.00216663	0.39799733	0.00687402	0.05384594	0.18190798	0.00373258
SLFL	0.00444538	0.35392143	0.00527617	0.000531	0.47673339	0.0036329
SLFR	0.01465996	0.27504373	0.00449986	0.01806566	0.26054001	0.00287125
UNCL	0.10039371	0.1356746	0.00205944	0.03977016	0.20413202	0.00301418
UNCR	0.01978177	0.25417942	0.00248636	0.12265305	0.12079099	0.00205944
VOFL	0.00753326	0.31991458	0.00403512	0.01647233	0.26697471	0.00334213
VOFR	0.00179929	0.40896507	0.00432513	0.00824688	0.31394034	0.00391899
pAFL	0.00363345	0.36655449	0.00514678	0.00389854	0.36216688	0.00321604
pAFR	0.00976442	0.30268367	0.00439779	0.008136	0.31483634	0.00302265

Table S2: **Relationship between gestational age and R1 in each white matter bundle (WM) and their gray matter (GM) targets.** The table summarizes the statistical relationships between scan age (gestational age at measurement in weeks) and R1 in each bundle. For each bundle, the correlation (r^2), the coefficient (slope), and the associated p-value is reported separately for white matter (WM) and gray matter (GM). Statistically significant correlations ($p < 0.05$) are highlighted in green. Abbreviations: GM: gray matter, WM: white matter, AF: Arcuate Fasciculus, ATR: Anterior Thalamic Radiation], CC: Cingulum Cingulate, CS: Cortico-Spinal Tract, FcMa: Forceps Major, FcMi: Forceps Minor, IFO: Inferior Frontal Occipital Fasciculus, ILF: Inferior Longitudinal Fasciculus, MLF: Middle Longitudinal Fasciculus, OR: Optic Radiation, SLF: Superior Longitudinal Fasciculus, UNC: Uncinate Fasciculus, VOF: Ventral Occipital Fasciculus, pAF: Posterior Arcuate Fasciculus, L=left, R=right.

Methods clarity and quality control

5. How was quality of bundle identification assessed, especially in very preterm scans? Was quality related to age at scan, and could measurement noise vary across the relatively wide scan-age range? For tissue response function estimation: WM/GM components are strongly influenced by PMA at scan. It would be helpful to elaborate (in the methods, or here because I am curious) on how response functions were derived and whether age-related differences impacted tractography, especially in younger subjects. Were smaller or more complex bundles disproportionately affected?

Thank you for this clarifying question. We have now added a separate paragraph to the method section in which we clarify the quality assurance steps we undertook. In brief, bundle quality assessment was performed in an automated way for the large-scale dHCP data (by excluding all sessions where one or more white matter bundles contained 10 or fewer streamlines) and by manual visual inspection for the smaller-scale SVBP data. Quality assurance was performed in the same way for all ages. In addition, we edited our description of the tractography steps for clarity. Tractography was also performed similarly across all ages. Finally, we added two new supplementary figures in which we show example bundles from different ages from both the dHCP and the SVBP data, so that bundle quality across ages can be assessed. These figures, which are included below, show a consistent anatomical trajectory of bundles across ages. In the method section on page 16 lines 520-538 and lines 568-585, we now write:

Quality assurance

dHCP: For the dHCP data we implemented automated quality assurance steps suitable for a large data set. First, we excluded all sessions that exceeded two standard deviations from the mean with respect to absolute motion and the number of outlier slices replaced by FSL's eddy as in our prior work[59]. Eight additional sessions were excluded due to conspicuous image artifacts or alignment errors that were noted during data processing. Further, to ensure bundle quality, we excluded all sessions where one or more white matter bundles contained 10 or fewer streamlines, as in our prior work[59]. Overall 167 of 490 sessions were excluded due to low data quality.

SVBP: For the SVBP data we implemented a different set of quality assurance steps, as this smaller data set allowed for manual visual inspections of all data. First, consistent with prior work[4,5,95], we quantified the number of outlier slices replaced by FSL's eddy and excluded all sessions with more than 5% outlier slices. No sessions were excluded based on this criteria. Next, we visually inspected all dMRI and R1 data for quality, which led to the removal of six sessions due to conspicuous image artifacts or alignment errors. We also visually inspected all bundles of all individuals to confirm that they adhere to their expected anatomical trajectory. No sessions were excluded based on bundle quality issues. Overall, 6 of the 27 sessions were excluded due to data quality concerns.

Tractography

Diffusion MRI tractography was conducted similarly for the dHCP and SVBP data while also matching processing steps to our prior work with each of these data sets (dHCP[59], SVBP[5,95]). Using MRtrix3[110], first, tissue response functions were computed separately for white matter, gray matter, and CSF using the Dhollander algorithm[120]. We then computed fiber orientation distributions (FODs) with multi-shell multi-tissue constrained spherical deconvolution (CSD)[121] for the white matter and the CSF. The gray matter was not modeled separately, as the b-values between white and gray matter are not sufficiently different to allow a clean separation of the signals in early infancy. Multi-tissue informed log-domain intensity normalization was performed. A whole brain white matter connectome was then created for each session, and tractography was optimized using the tissue segmentation from anatomical MRI data (anatomically constrained tractography, ACT[122]), which is particularly useful when gray and white matter cannot be separated in the FODs. For each connectome,

we used probabilistic fiber tracking with the following parameters: number of streamlines: 2 million (dHCP) or 5 million (SVBP), algorithm: IFOD1 (dHCP) or IFOD 2 (SVBP), step size: 0.2 mm, minimum length: 4 mm, maximum length: 200 mm, FOD amplitude stopping criterion: 0.05, maximum angle: 15 deg. Seeds for tractography were randomly placed within the gray/white matter interface (from anatomical tissue segmentation), which enabled us to ensure that all streamlines reach the gray matter, which was critical for determining each bundle's cortical terminations.

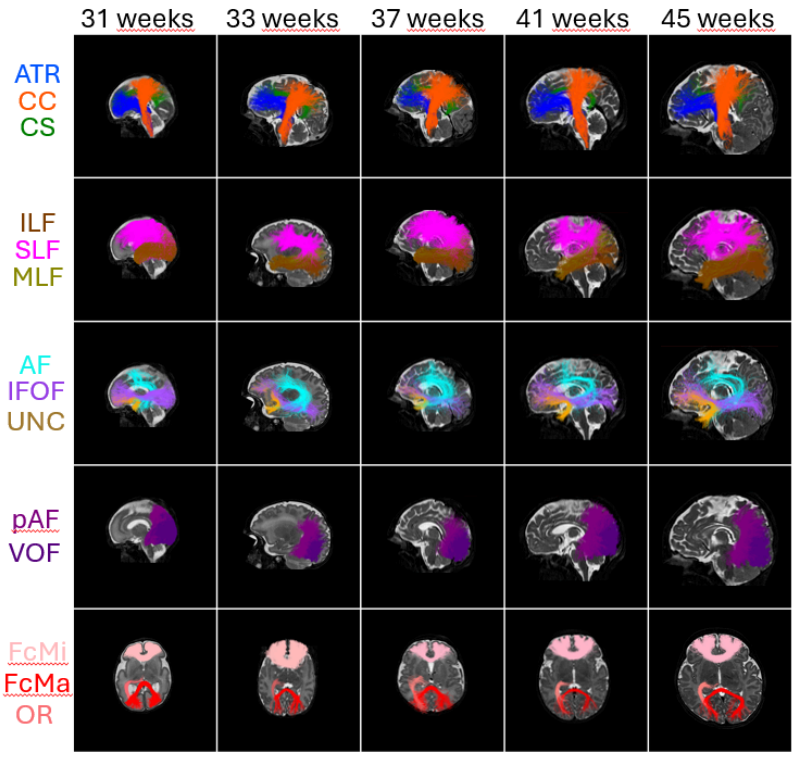

Figure S1: **Examples of bundles identified with pyBabyAFQ in the dHCP dataset.** Bundles are shown in nine randomly chosen individuals from dHCP scanned at gestational ages ranging from 31 weeks (left) to 45 weeks (right). Abbreviations: AF: arcuate fasciculus, ATR: anterior thalamic radiation, CS: cortico-spinal tract, CC: cingulum cingulate, FcMi: forceps minor, FcMa: forceps major, ILF: inferior longitudinal fasciculus, MLF: middle longitudinal fasciculus, IFOF: inferior frontal occipital fasciculus, OR: optic radiation, pAF: posterior arcuate fasciculus, SLF: superior longitudinal fasciculus, UNC: uncinate fasciculus, VOF: ventral occipital fasciculus.

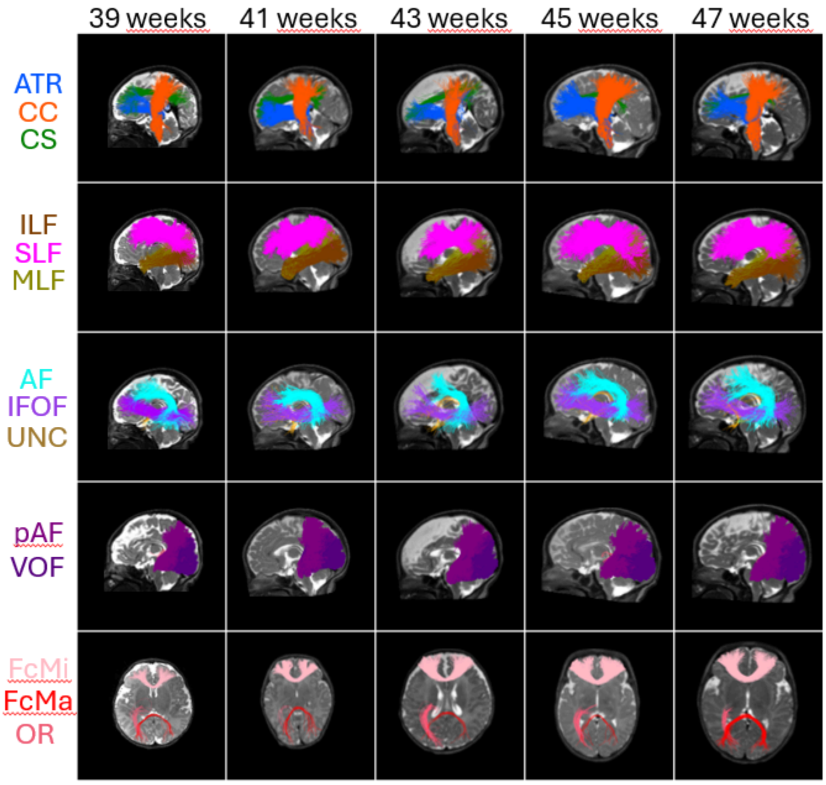

Figures S2: **Examples of bundles identified with pyBabyAFQ in the SVBP dataset.** Bundles are shown in five randomly chosen individuals from SVBP scanned at ages ranging from 39 weeks (left) to 47 weeks (right). Abbreviations: AF: arcuate fasciculus, ATR: anterior thalamic radiation, CS: cortico-spinal tract, CC: cingulum cingulate, FcMi: forceps minor, FcMa: forceps major, ILF: inferior longitudinal fasciculus, MLF: middle longitudinal fasciculus, IFOF: inferior frontal occipital fasciculus, OR: optic radiation, pAF: posterior arcuate fasciculus, SLF: superior longitudinal fasciculus, UNC: uncinate fasciculus, VOF: ventral occipital fasciculus.

6. Were cortical/subcortical delineations used for bundle endpoints checked against the surface parcellations? Would mismatches impact bundle–cortex coupling measures?

Thank you. We added this to the method section (on page 17, lines 604-606) where we write: "The same segmentations were employed for both bundle-endpoint identification and cortical-surface generation."

7. I am sorry, but upon reading it's hard to understand how bundle-wise GM/WM correlation within subject was computed. I thought you have one measure for GM and for WM per bundle per subject?

Thank you - you are correct, we have only one measure for GM and for WM per bundle per subject and we therefore do not present bundle-wise GM/WM correlation within subjects. Are you perhaps referring to figure 3a-b? In this figure, we are plotting T1w/T2w of each subject relative to their scan age separately for each bundle and tissue. In other words, here we are not plotting the correlation across tissues but rather just the underlying T1w/T2w values in each tissue and bundle that was used to compute the developmental slopes.

8. Wondering if there was any correction for multiple comparisons throughout the paper, I assume yes but it would be good to state explicitly what was corrected for what when you are presenting the results.

Yes - we use Bonferroni correction where applicable and in particular when relating the T1w/T2w correlations to behavioral outcomes, as multiple tests were run in that analysis. We describe this in the method section (on page 19 lines 662-673) where we write:

"Next we tested if these inter-individual differences have behavioral consequences. For this, we used a subsample of 215 infants which also completed the Bayley Scales of Infant and Toddler Development [...] For each measure, Pearson's correlations were used to assess associations. Bonferroni correction was applied. "

Prematurity and timing effects

9. Was prematurity related to time between birth and scan? (for very early preterms it might be difficult to scan close to birth). Would this interact with your observations?

Thank you. The time between birth and scan was indeed slightly different between infants born preterm (mean 2.97, SE 3.47) and full-term (mean 1.03, SE 1.19), which impacts our observations in so far that it highlights the importance of looking at both factors, an infant's birth age and their scan age, and to evaluate how these two factors impact the T1w/T2w coupling. We explicitly evaluate the impact of prematurity and the time between birth and scan on the coupled development of gray at white matter in the analyses related to Figure 5 included below. In brief, we find reduced T1w/T2w coupling across tissues in preterm infants at birth and even once they reach term-equivalent age. Please note that the result at term-aquivalent age has changed compared to our prior submission, as we noticed an error in the matching procedure that was now corrected. In addition to this analysis that was done in a small longitudinal subsample, we now also tested the impact of gestational age at scan

and gestational age at birth on the T1w/T2w coupling in the entire dHCP sample and, as shown in figure 3 included below, find that both of these factors impact the T1w/T2w synchrony. Taken together, these results suggest that the T1w/T2w coupling increases with increasing brain maturity.

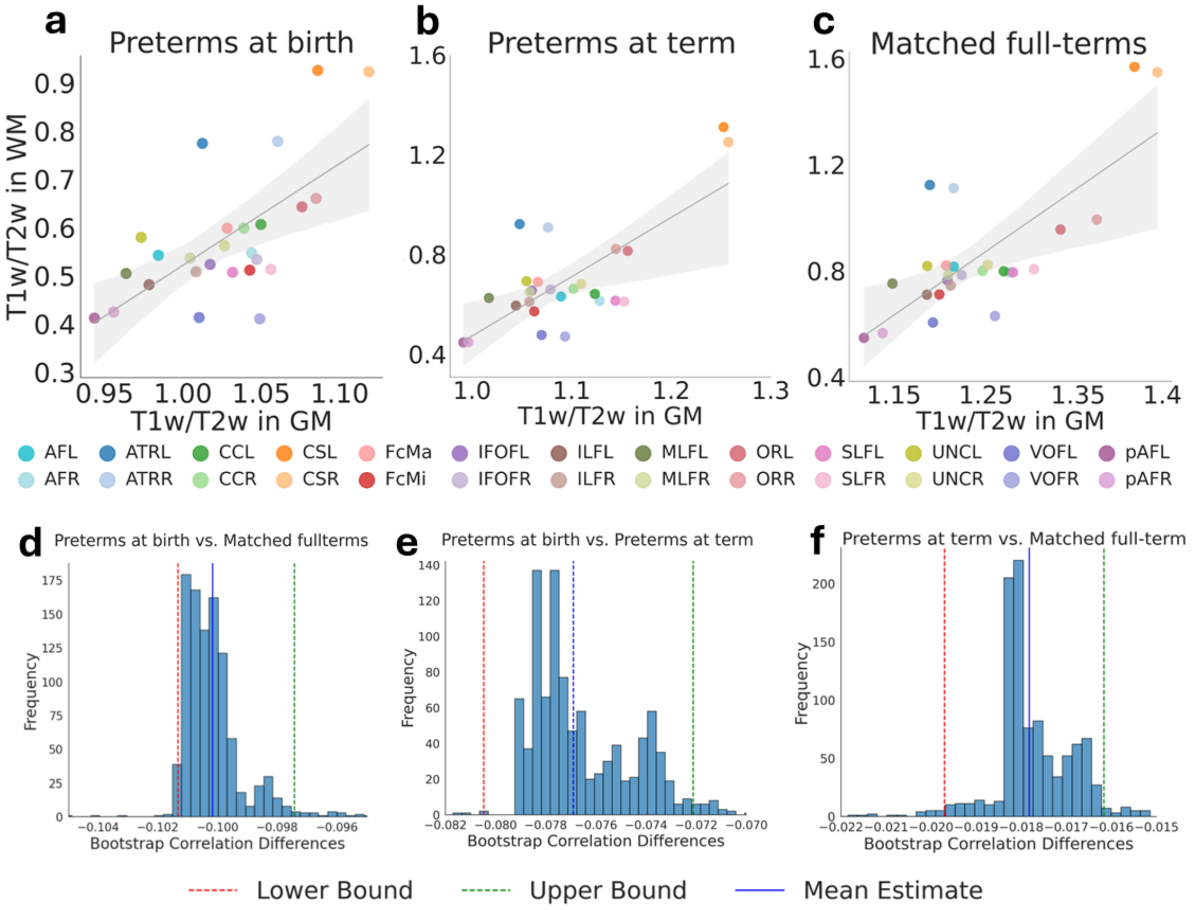

Figure 5: **Prematurity impacts the T1w/T2w coupling across tissues**. a-c Correlation of T1w/T2w across tissues in three groups: a infants born preterm scanned shortly after birth (r^2=0.43, p-value=0.0002); **b** infants born preterm scanned at term-equivalent age (i.e. at ≥ 37 weeks gestational age, r^2=0.54, p<0.0001); **c** age-matched infants born full-term (r^2 = 0.58; p<0.0001). Each dot is a bundle. **d-f** Bootstrap distributions of correlation differences between groups: **d** preterm infants scanned shortly after birth vs. full-term infants; **e** preterm infants scanned shortly after birth vs. preterm infants scanned at term-equivalent age; **f** preterm infants scanned at term-equivalent age vs. full-term infants. The vertical red and green lines indicate the upper and lower bound of the 95% confidence intervals (CIs), blue line indicates mean. Zero fell outside the 95% CIs in all comparisons. Abbreviations: AF: Arcuate Fasciculus, ATR: Anterior Thalamic Radiation, CC: Cingulum Cingulate, CS: Cortico-Spinal Tract, FcMa: Forceps Major, FcMi: Forceps Minor, IFOF: Inferior Frontal Occipital Fasciculus, ILF: Inferior Longitudinal Fasciculus, MLF: Middle Longitudinal Fasciculus, OR: Optic Radiation, SLF: Superior Longitudinal Fasciculus, UNC: Uncinate Fasciculus, VOF: Ventral Occipital Fasciculus, pAF: Posterior Arcuate Fasciculus, L=left, R=right.

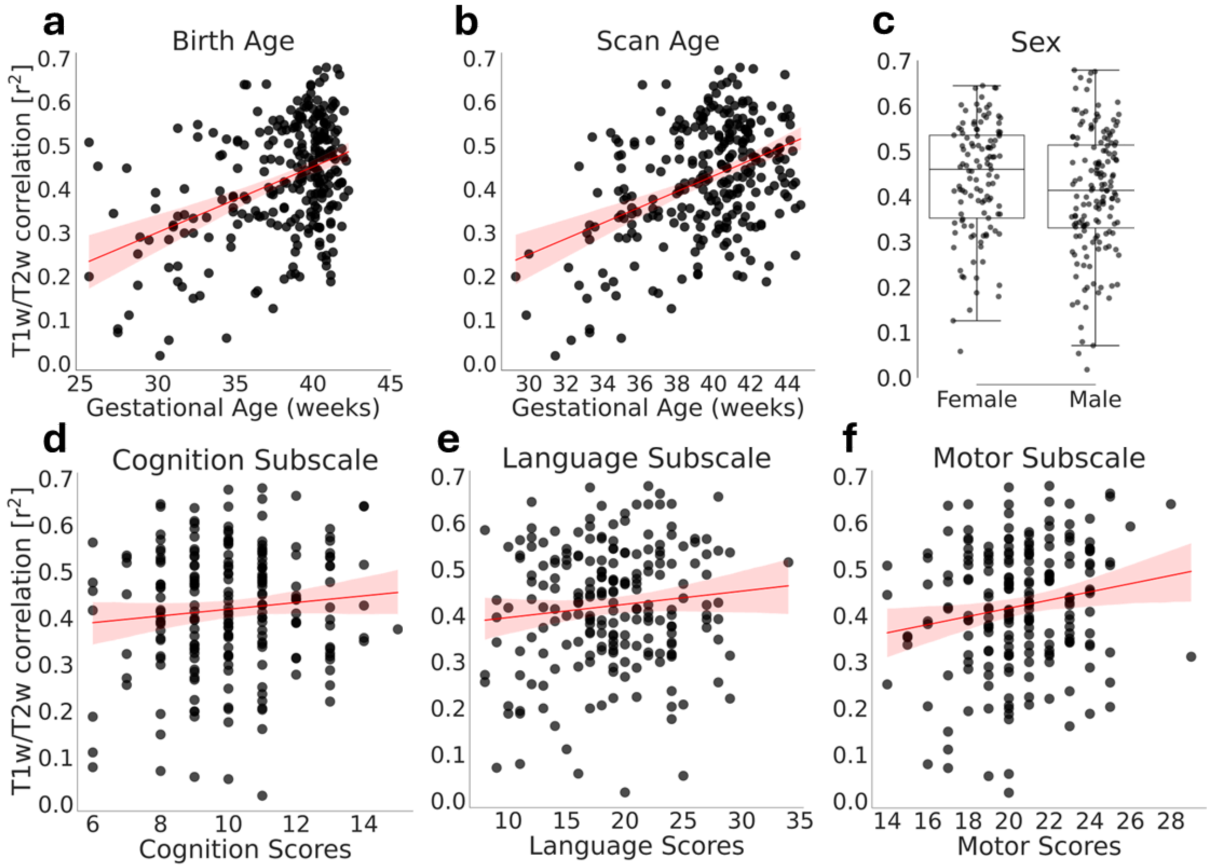

Figure 4: **Characterization of inter-individual differences in the T1w/T2w coupling and its relationship to behavior. a-c** Upper row relates T1w/T2w coupling across tissues to gestational age at birth (a, r^2=0.18, p-value=$5.15e^{-13}$), gestational age at scan (b, r^2=0.18, p-value=$2.23e^{-13}$), and sex (c, t=-1.88, p-value=0.06). **d-f** Bottom row relates the correlation of T1w/T2w in gray and white matter to the cognition (d; r^2=0.01, p-value=0.14), language (e; r^2=0.01, p-value=0.11), and motor (f; r^2=0.03, p-value=0.02) subscale of the Bayley-III. We found a relationship between the T1w/T2w coupling and motor skills, but not the other subscales. Each dot is a subject, red lines indicate linear regression lines, shaded regions indicate 95% confidence interval, boxes in c indicate the interquartile range (IQR, 25th–75th percentile), the line inside each box denotes the median and the whiskers extend to 1.5×IQR.

10. I am struggling to interpret the results in the section “T1w/T2w coupling across tissues is linked to infants’ motor skills later in life” and the related conclusion (iv). The analyses appear to rely on a cross-sectional sample spanning a wide developmental age range. For this period, both age at scan and GA at birth should be carefully controlled when relating imaging measures to later outcomes (and age at assessment as well, if raw Bayley scores were used, as mentioned in the methods). This is especially important given your other conclusion that “In measurements conducted shortly after birth, coupling between T1w/T2w in white and gray matter is weaker at preterm than at full-term birth”. In other words, what may appear as a coupling–outcome association could instead reflect the effect of prematurity: infants scanned earlier (more premature) both show lower scores and weaker coupling, without coupling itself being the factor. To support the claim that lower coupling per se predicts lower motor outcomes, the analyses should at minimum adjust for age at scan (and ideally GA at birth). Even better, you could restrict the analysis to infants scanned at term (>37 wGA) to reduce this confound. I would also strongly recommend plotting GA at birth and/or scan against Bayley scores to clarify this relationship. As currently framed, the

conclusion that “higher coupling predicts better motor skills” seems insufficiently justified without these adjustments.

Thank you for this comment. First, please also note that we actually evaluated age-standardized rather than raw Bayley scores - this was incorrectly stated in the prior submission, we apologies for the oversight. To further address this concern, we evaluated the effect of scan age and birth age on the T1w/T2w coupling. We found that your assumption that these factors impact T1w/T2w synchrony is indeed correct, as can be seen in Figure 4 included above. To address your concern, we performed additional model comparison analyses to formally test whether age at scan, gestational age at birth, or sex contributed to the prediction of motor outcomes beyond the T1w/T2w coupling (r^2). Specifically, we compared a baseline model (*motor score* ~ r^2) to three extended models that each included one additional covariate (scan age, birth age, or sex). None of the extended models provided a significant improvement in model fit relative to the baseline model. If gestational age was the main predictor of motor skill, we would have expected that adding this factor would improve the model, nonetheless as T1w/T2w coupling and gestational age were correlated, we have toned down our conclusions regarding the impact of this coupling on behavior.

In the discussion, on page 13 lines 394-399, we now write: “Our findings complement this literature by showing that it may not only be the absolute amount of myelin that impacts motor performance, but also the degree of synchrony in myelination across white matter bundles and their corresponding gray matter targets. However, as the observed impact of T1w/T2w coupling on motor performance is comparatively small and the coupling itself is impacted by gestational age, future work is needed to assess the implications of this effect. ”

11. ‘This suggests that the link between T1w/T2w across tissues is dependent on brain maturity as well as on experience. After all, at birth, the brains of preterm infants had less time to mature compared to their full-term born peers, which could explain the observed reduced T1w/T2w coupling. Indeed, research indicates that preterm birth can alter both the speed and degree of brain myelination, potentially leading to developmental differences when compared to full-term infants’ This is strangely worded. Reduced coupling shortly after preterm birth compared to full-term could simply reflect typical maturational differences, as 34wGA brains are less mature than 40wGA. Comparing preterm infants at TEA to full-term neonates is the true test of prematurity effects.

We agree and have edited this paragraph for clarity and also to incorporate the new results where we tested the effect of birth age and scan age across the entire dHCP sample. In the discussion, on page 13 lines 413-423 we now write: “Our data suggest that the synchrony of gray and white matter myelination may be dependent on brain maturity. First, we find that both gestational age at birth and gestational age at scan are positively related to inter-individual differences in the degree of T1w/T2w coupling across tissues. Further, in a longitudinal sub-sample, we find lower correlation of T1w/T2w across gray and white matter in preterm compared to full-term infants at birth.. Interestingly, even at term-equivalent age, when the age-matched preterm and full-term born infants have had the same amount of time to mature, they still show different degrees of T1w/T2w coupling with coupling being stronger in the full-term born infants. These results are consistent with prior work indicating that

preterm birth can alter both the speed and degree of brain myelination, potentially leading to developmental differences when compared to full-term infants[59,104,105] (for review see[106]).”

12. The discussion of sensory experience in preterm infants could benefit from reframing. Rather than simply receiving “more” input, these infants experience qualitatively different, often noxious, sensory stimulation, which may dysregulate development (in contrast to typical in-utero development which has plenty of sensory experience, which is necessary for optimal maturation). Stronger T1w/T2w coupling does not necessarily indicate “better” outcomes (this is also important in context of ‘seemingly surprising relationship between coupling and later outcomes versus prematurity’ that you mention in the discussion) especially without taking into account the wide age range in the cross-sectional sample which I suggested above might be interacting with the outcome results.

Thank you. As laid out in detail in response to your comment right above, we sharpened the paragraph of the discussion pertaining to the effects of premature birth and in that process also removed all discussion of their sensory input. We included the updated paragraph above.

Analyses and figures

13. Figure 2: please indicate ages at scan for subjects B, C, D. Could apparent correlations reflect age or bundle quality?

Thank you. We have added GA at scan of the example subjects to the figure caption of Figure 2. As these randomly chosen participants may not be representative of the entire sample, we also explicitly tested the effect of GA at scan on the T1w/T2w coupling and found that T1w/T2w coupling is impacted by GA at scan. These new results are presented in figure 4 included above.

14. Correlation coefficients are sensitive to variance: bundles with narrow T1w/T2w ranges will give lower r. It is difficult to compare the results across bundles without visualizing distributions. A boxplot or similar visualization of raw T1w/T2w values across bundles would be very helpful. In the same vein, also during typical development, if we assume there are no methodological artefacts related to GA, one might expect lower individual variability that increases with age, would this play a role in estimation of ‘lower coupling’ at earlier ages?

Thank you. We have added boxplots of the raw T1w/T2w (and R1) values across bundles to the supplement (Supplementary Figure S5) and are including this figure below. We don’t expect that inter-bundle variance contributes to the results as our main effect of T1w/T2w coupling is observed across bundles and not within a given bundle. Also when evaluating new Figures 4a and 4b, we don’t see an effect of individual variability contributing to the increased coupling with increasing scan and birth age.

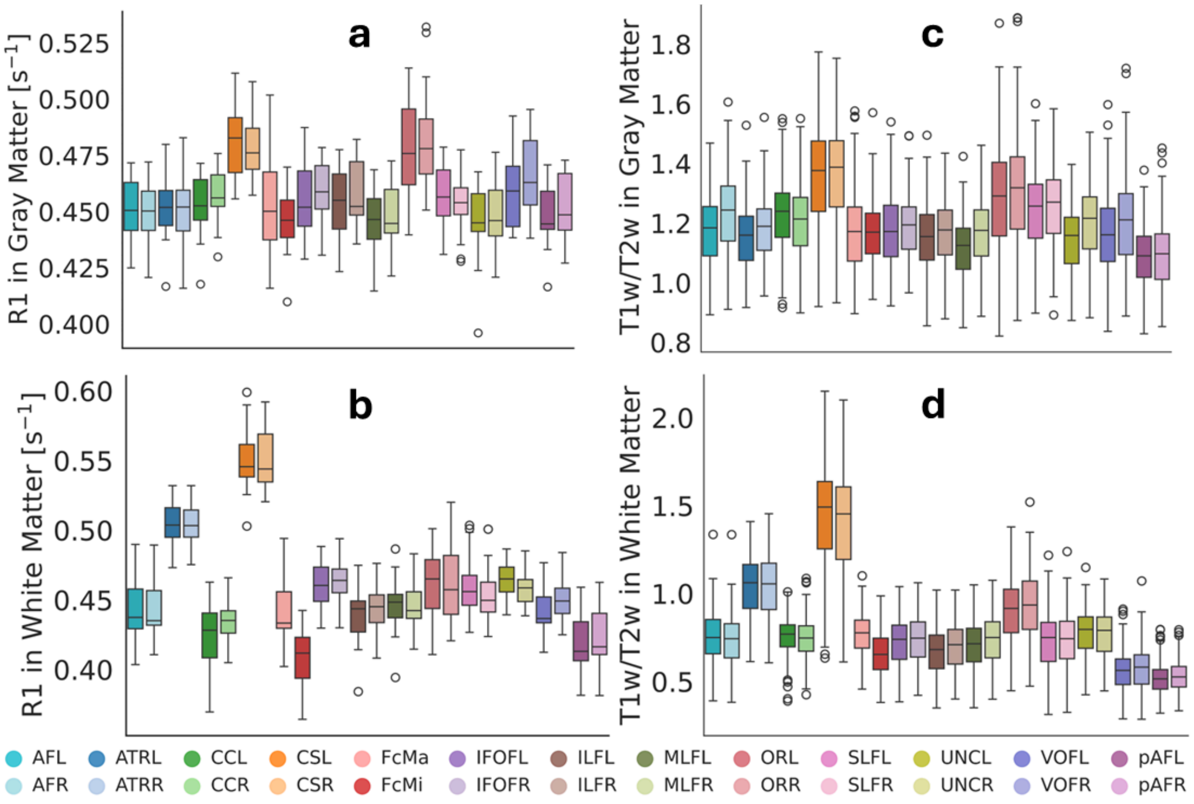

Figure S5: **Boxplots showing differences in T1w/T2w and R1 in white and gray matter across bundles. a,b** R1 in gray matter (a) and white matter (b), respectively. **c,d** T1w/T2w in gray matter (c) and white matter (d) respectively. Each box represents the interquartile range with median (horizontal line), whiskers indicating 1.5 times the interquartile range, and points representing outliers. Colors indicate bundles. Abbreviations: AF: Arcuate Fasciculus, ATR: Anterior Thalamic Radiation], CC: Cingulum Cingulate, CS: Cortico-Spinal Tract, FcMa: Forceps Major, FcMi: Forceps Minor, IFO: Inferior Frontal Occipital Fasciculus, ILF: Inferior Longitudinal Fasciculus, MLF: Middle Longitudinal Fasciculus, OR: Optic Radiation, SLF: Superior Longitudinal Fasciculus, UNC: Uncinate Fasciculus, VOF: Ventral Occipital Fasciculus, pAF: Posterior Arcuate Fasciculus, L=left, R=right.

15. Figure 5: does the difference between panels B and C largely reflect CCL/CCR? Is there any reason why you think CC diverges so much between the two groups?

We believe the bundle you are referring to is the CSL (orange), the bundle legend was a bit misleading before, which we have also fixed. The difference between panels B and C is not solely driven by the CS. Even when we remove this bundle, zero remains outside of the 95% confidence intervals of the bootstrapped correlation differences as indicated in the figure below.

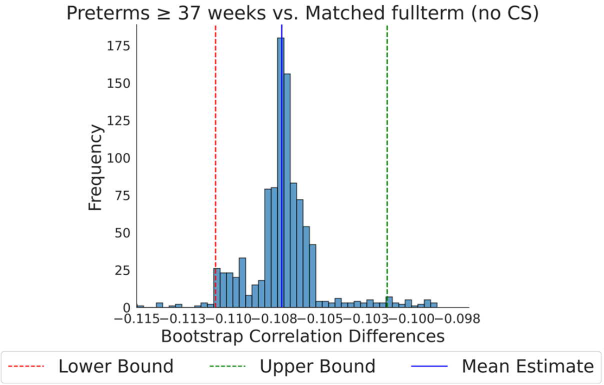

Bootstrap distribution of correlation differences between groups, excluding cortico-spinal tract (CS) in both groups: preterm infants scanned at term-equivalent age vs. full-term infants. The vertical red and green lines indicate the upper and lower bound of the 95% confidence intervals (CIs), blue line indicates mean. Zero fell outside the 95% CIs in all comparisons.

16. Bayley-III: Please justify using raw score sums rather than age-corrected composite or scaled scores. Raw scores are not age-adjusted and could confound developmental interpretations to my knowledge?
Our apologies, we actually are using age-adjusted scores, this was incorrectly stated in the initial submission and has been corrected. In the method section page 19 lines 667-671 we now state "We examined three behavioral domains (cognition (N=209), language (N=210), and motor (N=205)) by taking the sum of age-standardized scores within each respective Bayley-III subscale and related these behavioral outcomes to the correlation of T1w/T2w across white and gray matter at birth (Fig. 4d-e)."

Minor comments

• '(GA at birth: mean SD: 32.04 ± 2.97 weeks)' -> '(GA at birth: mean ± SD: 32.04 ± 2.97 weeks)'
We corrected this.
• 'GA of at least 37 weeks (GA at second scan…)' you refer to this groups as 'preterm infants scanned at term-equivalent age' later on, it would be helpful to homogenise across the manuscript
We homogenized this.
• "Overall, these results suggest that white and gray matter are developing in concert during early infancy. ." → remove extra period.
We corrected this.
• Matter of personal taste, Figure 3: consider omitting regression lines for nonsignificant relationships as this might be confusing at a glance. Showing thresholds of atypicality for Bayley scores would contextualize results, since most toddlers within dHCP perform within the typical range (although I am not sure this is possible with raw scores).
We decided to keep the regression lines as we feel that even for non-significant results it can be helpful to see the directionality of regression output.
• It might make figures more readable if you used same colour for bilateral bundles but used a different shape for left, and right. This would significantly simplify the colour scheme making it easier to identify bundles on scatter plots.
We decided to keep the color scheme, as we are always using the same color scheme across all our studies evaluating white matter bundles. We feel that this makes it easier to quickly compare results across studies.
• Discussion: reads well, but should be tightened for clarity and focus (see analysis-related comments). Similarly, the data section can be shortened, no need for extensive background on dHCP beyond what data were used.
We have substantially tightened the discussion in response to your feedback. We got somewhat contradictory feedback across reviewers whether more or less background on the dHCP data should be included in the method section, so we sharpened the method section while at the same time ensuring that all necessary information is presented.

I hope my comments are helpful in refining the manuscript and clarifying the analyses.
I am looking forward your responses,
Andrea Gondova

Thank you again for your feedback which we feel has enabled us to substantially improve the manuscript.

Reviewer #2

This study examines relationships between white and cortical gray matter T1w/T2w in a large cohort of infants from the Developing Human Connectome. In particular, the authors investigated the degree to which the white and cortical gray matter T1w/T2w are inter-related across a number of white matter bundles, how these are shaped by early development, and if the T1w/T2w correlation/coupling is related to later infant behavior/cognition. The authors observe both the mean and rates of changes of the T1w/T2w of white matter bundles to be correlated with the T1w/T2w of the cortical "targets" and the correlation between T1w/T2w white and gray matter associated with improved motor skills later in childhood. The authors also compare these relationships/patterns between preterm, preterm at term-equivalent-age (TEA) and full-term infants, observing the correlations to be higher in the TEA infants than the full-term. The authors argue that these patterns highlight that the changes in T1w/T2w in early infancy are strongly white and gray matter. Overall, the authors perform an interesting analysis on a large infant sample. However, I have several questions around the method and analyses performed that should be clarified. The paper is also quite dense and long and I think there are areas where the text should be clarified and/or reduced.

Thank you for your detailed evaluation, which helped us to improve our manuscript. First, in regards to your comment and a similar comment from reviewer 1, we have sharpened the manuscript, particularly in the introduction and discussion and edited the manuscript for clarity. We also have implemented several additional changes in response to your comments, which we detail below.

Comments:

1) While the dHCP pipelines were used for the preprocessing of data, it would be useful/informative to include additional information about these steps. For example, was the bias field correction performed separately on the T1w and T2w images? How do these steps effect the T1w/T2w ratios? Was the registration between the T1w and T2w images a rigid-body registration or were more degrees of freedom used?

Thank you. We got contradictory feedback on how much detail we should provide for the dHCP pipelines. In fact, reviewer 1 requested the exact opposite and asked us to remove all details about the dHCP processing pipelines from our method section. As there are several publications from the dHCP in which their pipelines are described in great detail (the T1w/T2w map creation and broader structural pipeline is described in Makropoulos et al. 2018), we felt a reasonable compromise would be to provide only a brief summary. In the method section, on page 15, lines 492-501, where we write:

dHCP: The anatomical and diffusion-weighted data used in this project underwent several preprocessing steps as part of the dHCP preprocessing pipelines[107–109] and here we used the preprocessed data. As accurate alignment of dMRI and anatomical data was critical for this project, we applied an additional rigid-body alignment using mrregister (from MRrtrix3[110]) and visually inspected all images for alignment quality. We also used the T1w/T2w maps provided by the dHCP[111]. Information on the generation of these maps are provided directly by the dHCP[112]; very briefly, the T1w image was first rigidly registered to the T2w image and the T1w/T2w was then estimated from the original T2w image and the transformed T1w

image (prior to bias correction). Following this, the T1/T2 ratio was projected onto the midthickness surface, using volume-to-surface mapping.”

2) How are intensity differences between subjects controlled for or handled with the T1w/T2w ratio. Specifically, given that different scanner settings will influence the individual intensities of a given T1w or T2w image (e.g. primary reason why one cannot compare T1w or T2w intensities across subjects), its not clear how just the ratio between T1w and T2w images accounts for such differences. For example, Ganzetti et al., (Ganzetti et al., 2014) describe an ‘intensity standardization’ procedure after bias correction for computing T1w/T2w ratios to help standardize values across subjects. Is a similar standardization performed here or how

Here we used the T1w/T2w ratio maps as provided by the dHCP without any additional pre- or post-processing. We feel that this is beneficial, as it allows for results to be easily replicated with these publicly available maps. Nonetheless, we acknowledge that the optimal way of computing T1w/T2w is debated and more generally that the usage of T1w/T2w as a proxy for myelin requires further validation. As such, we have now added a complementary quantitative neuroscience data set that, in addition to dMRI also includes qMRI data, and hence enabled analyses of R1. Quantitative R1 is widely accepted as a proxy for myelin and its direct relationship with myelin content has been confirmed in post-mortem data (see e.g. Schüber el al., Neuroimage, 2014). Using these complementary data we found striking consensus between T1w/T2w and quantitative R1. Most importantly, as shown in new figure 2 and 3 included below, both T1w/T2w and R1 show correlated mean values and developmental slopes across tissues. The newly added R1 analyses hence further supports our claim of coupled myelin development across tissues with a more widely accepted and validated measure. These new results are presented in the first and second paragraph of the results section.

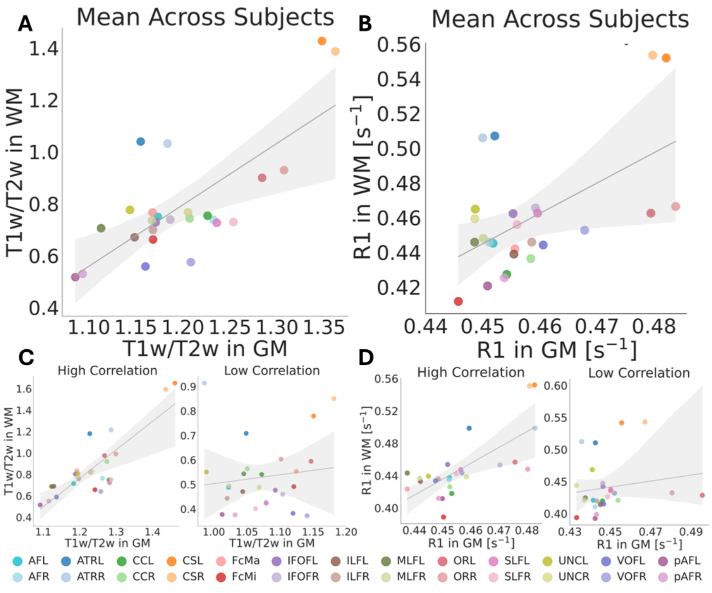

Figure 2. **Relationship between T1w/T2w and R1 of WM bundles and their respective GM targets.** Each dot is a bundle. **a** Mean correlation with T1w/T2w values averaged across subjects (r^2=0.55, p-value=$1.45e^{-5}$). **b** Mean correlation with R1 values averaged across subjects (r^2=0.32, p-value=0.002). **c** Example individual subjects exhibiting high (left; GA at scan: 41.43 weeks; r^2=0.68, p-value=$2.28e^{-7}$) and low (right; GA at scan: 31.43 weeks; r^2=0.017, p-value=0.52) correlation between T1w/T2w of white and gray matter. **d** Example individual subjects exhibiting high (left; GA at scan: 42.5 weeks; r^2=0.51, p-value=$4.21e^{-5}$) and low (right; GA at scan: 40.4 weeks; r^2=0.03, p-value=0.44) correlation between R1 of white and gray matter. Abbreviations: WM: white matter, GM: gray matter, AF: Arcuate Fasciculus, ATR: Anterior Thalamic Radiation, CC: Cingulum Cingulate, CS: Cortico-Spinal Tract, FcMa: Forceps Major, FcMi: Forceps Minor, IFOF: Inferior Frontal Occipital Fasciculus, ILF: Inferior Longitudinal Fasciculus, MLF: Middle

Longitudinal Fasciculus, OR: Optic Radiation, SLF: Superior Longitudinal Fasciculus, UNC: Uncinate Fasciculus, VOF: Ventral Occipital Fasciculus, pAF: Posterior Arcuate Fasciculus, L=left, R=right.

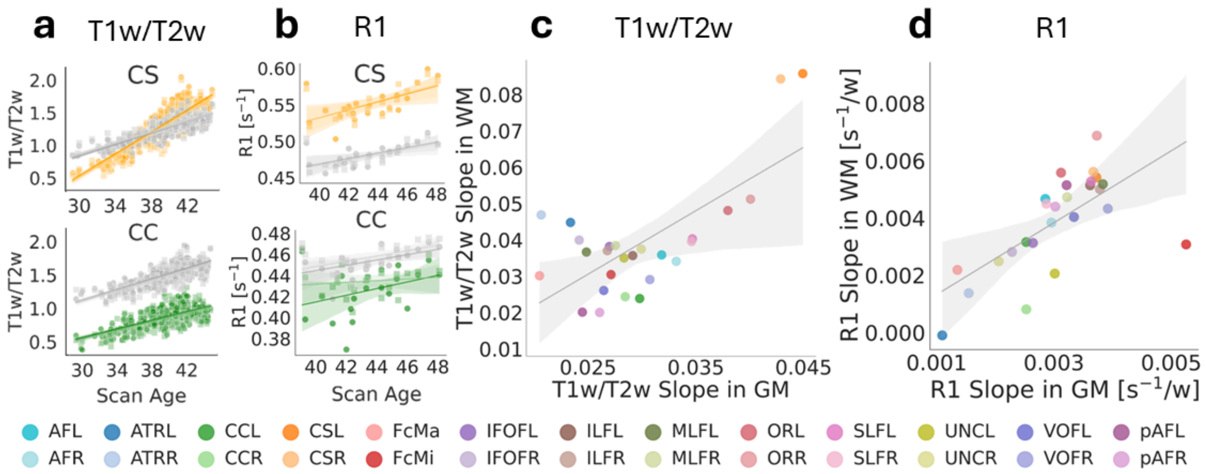

Figure 3: **The rates of change of myelin-sensitive imaging metrics are correlated across white and gray matter. a,b** Two example bundles (cortico-spinal tract (CS, top) and cingulum cingulate (CC, bottom)) illustrate T1w/T2w (a) and R1 (b) slopes in gray and white matter (for all bundles see Supplementary Figures S6 and S7). Gray circles indicate gray matter values in each subject, colored circles represent white matter values in each subject, and the steepness of the lines indicate the slopes (rates of change relative to the infant's gestational age at the time of measurement in weeks), the two hemispheres are presented in different shades. **c,d** Relationship between the slopes of T1w/t2w (c) and R1 (d) along white matter (WM) bundles and their gray matter (GM) targets (T1w/T2w: r^2=0.49, p-value=6.28e^{-5}; R1: r^2=0.45, p-value=0.0002). Each circle indicates a bundle, the two hemispheres are presented in different shades. Abbreviations: AF: Arcuate Fasciculus, ATR: Anterior Thalamic Radiation, CC: Cingulum Cingulate, CS: Cortico-Spinal Tract, FcMa: Forceps Major, FcMi: Forceps Minor, IFOF: Inferior Frontal Occipital Fasciculus, ILF: Inferior Longitudinal Fasciculus, MLF: Middle Longitudinal Fasciculus, OR: Optic Radiation, SLF: Superior Longitudinal Fasciculus, UNC: Uncinate Fasciculus, VOF: Ventral Occipital Fasciculus, pAF: Posterior Arcuate Fasciculus, L=left, R=right.

3) The authors should be careful to refer to the T1w/T2w ratio as a measure of myelination. As they acknowledge, no MR measure directly measures myelin and other quantitative imaging techniques, like relaxometry/myelin water fraction imaging, or quantitative magnetization transfer imaging methods, appear more sensitive to myelin content than the T1w/T2w ratio (see Uddin et al., 2018; 2019). Moreover, while R1 is more sensitive to myelin, the statement that because R1 is higher in the cortex in infants there is more myelin content in the cortex seems inaccurate. When examining other studies using myelin water imaging or magnetization transfer techniques, this is not the case. Moreover, while R1/qT1 is sensitive to myelin, there are a number of other things that directly influence R1 (cytoarchitecture, water content, etc…).

Thank you for this comment. We agree that the validity of using T1w/T2w as a marker for myelin requires further evaluations and have therefore now added analyses of R1 to the manuscript. We could successfully replicate the coupled development of white and gray matter properties with this more sensitive measure. Moreover, we have removed the statement that "because R1 is higher in the cortex in infants there is more myelin content in the cortex" from the manuscript.

4) What was the purpose for performing Tractography when the pyBabyAFQ is being used to generate the white matter bundles? This was unclear in how this is being used. Was it for the cortical terminations? If so, was it ensured that the cortical terminations were consistent with the bundles from the pyBabyAFQ?

Thank you for these clarifying questions. We chose to perform tractography with MRtrix rather than AFQ as AFQ currently does not support anatomically-constrained tractography (ACT), which we have found to be beneficial for the infant brain in which there is a low and inconsistent difference in FA between gray and white matter. As such we performed tractography in MRtrix3 and then “fed” the generated whole-brain tractogram into pyBabyAFQ for bundle delineation. Only once the bundles were delineated in pyBabyAFQ did we determine their cortical termination, so the cortical terminations were derived from and hence consistent with the bundles from pyBabyAFQ. We have edited the respective paragraphs of the method section for clarity.

5) Presumably the diffusion MRI data need to be registered to the anatomical data to be able to extract the T1w/T2w from the bundles. Was this performed as part of the dHCP pipelines or how was this done?

Yes, this was performed as part of the dHCP pipeline. To ensure accurate registration, we performed an additional rigid-body alignment using mrregister (from MRrtrix3) and we visually inspected all images for alignment quality. We have now added this information to the method section, where we write on page 15, lines 492-496:

dHCP: The anatomical and diffusion-weighted data used in this project underwent several preprocessing steps as part of the dHCP preprocessing pipelines[107–109] and here we used the preprocessed data. As accurate alignment of dMRI and anatomical data was critical for this project, we applied an additional rigid-body alignment using mrregister (from MRrtrix3[110]) and visually inspected all images for alignment quality.

6) I would suggest creating a figure that illustrates the process for generating the white matter and cortical T1w/T2w measures.

Thank you. We have now included an additional figure in the main manuscript that provides an overview of our pipeline. This new Figure 6 is included below.

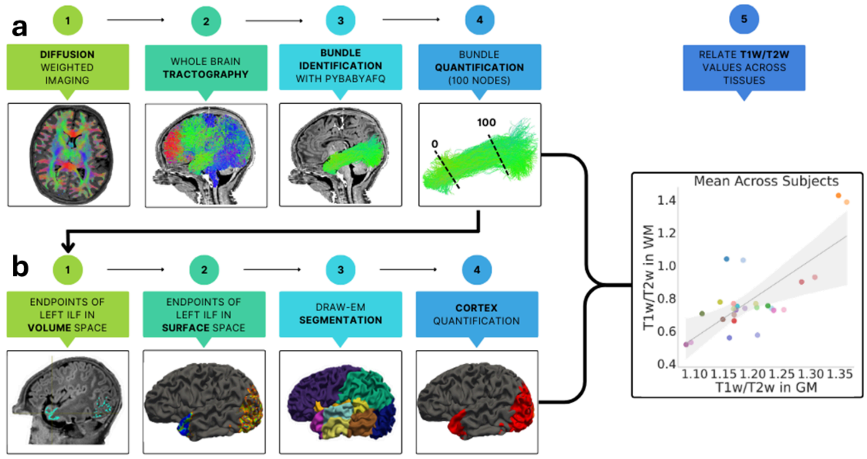

Figure 6: **Workflow to obtain white matter (a) and cortical (b) myelin-sensitive imaging metrics**. The T1w/T2w of the left inferior longitudinal fasciculus (ILF) is used as an example. White matter T1w/T2w values (top row): A whole brain tractogram is created from dMRI data (steps 1 and 2). Bundle identification is

performed with pyBabyAFQ (step 3). Each bundle is divided into 100 equidistant nodes and T1w/T2w is calculated at each node by taking a weighted average of each streamline's properties at that node (step 4). Gray matter T1w/T2w values (bottom row): After identifying the endpoints in volume (step 1) and surface space (step 2), two cortical endpoint ROIs from the draw-EM segmentation (step 3) serve as anatomical boundaries to separate terminations at the start and end of cortical-to-cortical bundles. At each termination, a weighted average of T1w/T2w is computed based on endpoint density in each voxel (step 4). Finally, T1w/T2w in gray and white matter are related to each other across all bundles (step 5).

7) In the discussion of the limitations, the authors state: "It is important to recognize, however, that the myelin content of infant white matter more closely resembles that of adult cortex than adult white matter, which has been shown by comparing R1 values across tissues from different age groups." I disagree with this sentence in comparing the infant white matter to the adult cortex and using this as justification. I also don't understand how this statement helps support the use of T1w/T2w in infants.

Thank you. We have removed this statement from the manuscript.

Reviewer #3

This interesting work looks at how a measure of tissue structure, the T1w/T2w ratio, changes concurrently with developmental age in both the white matter and the associated grey matter. It uses a set of known white matter bundles and their cortical targets to do this. It looks at the developmental trajectories of these associations and compares individual variability in how related these measures are within-subject to relate to outcomes at 18 months, and how they differ in prematurity.

It's a really nice way of looking at regional brain development but I've a few large concerns on how the data has been interpreted and how specific the age associations are.

Thank you for your feedback - we feel that your comments have enabled us to substantially improve our manuscript. We respond to each of your concerns in detail below.

Biology - There is a focus here on T1/T2 reflecting myelination. Even in adults, this value is criticised as not mapping to other (more validated) measures of myelination, especially in white matter (referenced by the authors here). In neonatal white matter in particular, outside of the primary sensory regions, the majority of signal change is more likely to be a reduction in water content as opposed to an increase in myelination. This is quite critical as the paper is very focused on testing a hypothesis of activity dependent myelination but I don't think this can be tested without one of the more specific white matter imaging techniques which can separate out the larger water signal. An example of a paper from this group (ref 59) using T1w/T2w in the same cohort showed that it is useable in this age group but not that it's specific to myelin.

Thank you for this important comment. We agree that the use of T1w/T2w as a proxy for myelin requires further validation. As such, we have now added a complementary quantitative neuroscience data set that, in addition to dMRI also includes qMRI data, and hence enabled analyses of R1. Quantitative R1 is widely accepted as a proxy for myelin and its direct relationship with myelin content has been confirmed in post-mortem data (see e.g. Schüber el al., Neuroimage, 2014). Using these complimentary data we found striking

consensus between T1w/T2w and quantitative R1. Most importantly, as shown in updated figure 2 and 3 included below, both T1w/T2w and R1 show correlated mean values and developmental slopes across tissues. The newly added R1 analyses hence further supports our claim of coupled myelin development across tissues with a more widely accepted and validated measure. These new results are presented in the first two paragraphs of the results section.

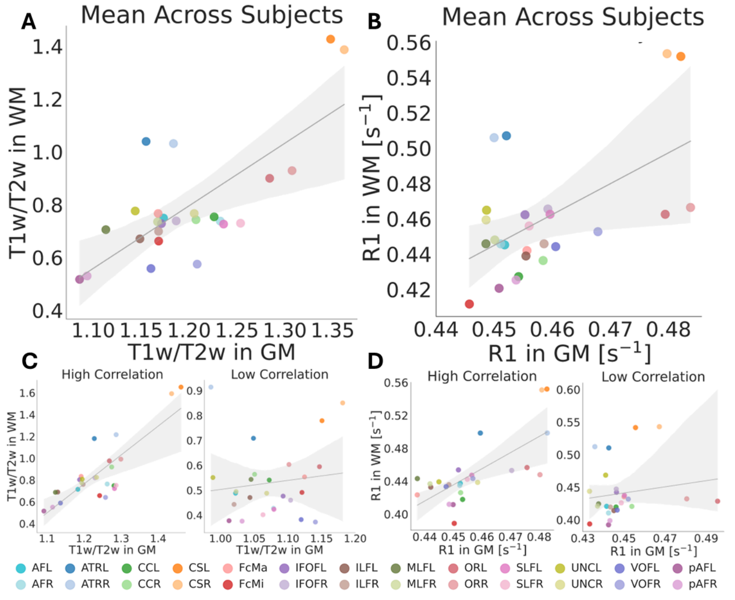

Figure 2. **Relationship between T1w/T2w and R1 of WM bundles and their respective GM targets.** Each dot is a bundle. **a** Mean correlation with T1w/T2w values averaged across subjects (r^2=0.55, p-value=1.45e^{-5}). **b** Mean correlation with R1 values averaged across subjects (r^2=0.32, p-value=0.002). **c** Example individual subjects exhibiting high (left; GA at scan: 41.43 weeks; r^2=0.68, p-value=2.28e^{-7}) and low (right; GA at scan: 31.43 weeks; r^2=0.017, p-value=0.52) correlation between T1w/T2w of white and gray matter. **d** Example individual subjects exhibiting high (left; GA at scan: 42.5 weeks; r^2=0.51, p-value=4.21e^{-5}) and low (right; GA at scan: 40.4 weeks; r^2=0.03, p-value=0.44) correlation between R1 of white and gray matter. Abbreviations: WM: white matter, GM: gray matter, AF: Arcuate Fasciculus, ATR: Anterior Thalamic Radiation, CC: Cingulum Cingulate, CS: Cortico-Spinal Tract, FcMa: Forceps Major, FcMi: Forceps Minor, IFOF: Inferior Frontal Occipital Fasciculus, ILF: Inferior Longitudinal Fasciculus, MLF: Middle Longitudinal Fasciculus, OR: Optic Radiation, SLF: Superior Longitudinal Fasciculus, UNC: Uncinate Fasciculus, VOF: Ventral Occipital Fasciculus, pAF: Posterior Arcuate Fasciculus, L=left, R=right.

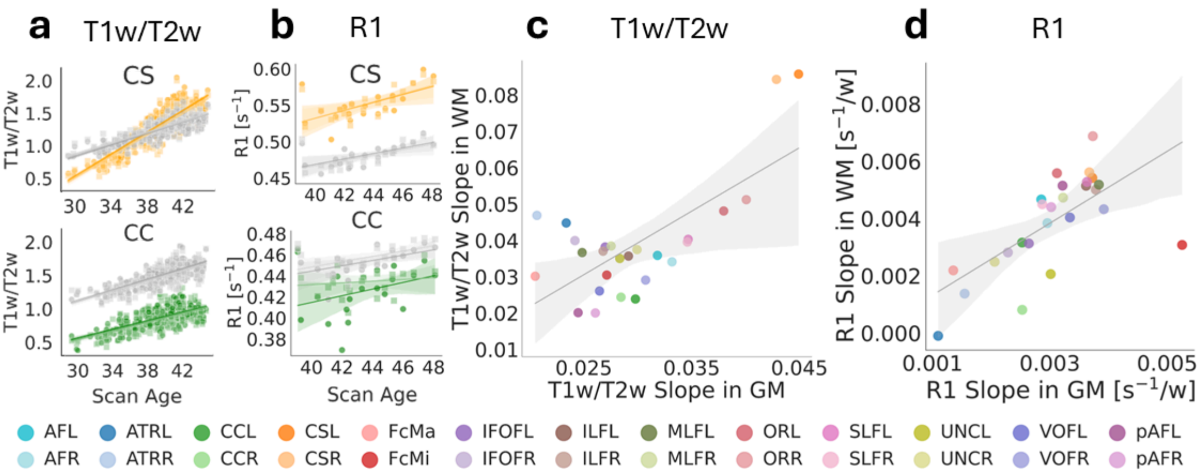

Figure 3: **The rates of change of myelin-sensitive imaging metrics are correlated across white and gray matter. a,b** Two example bundles (cortico-spinal tract (CS, top) and cingulum cingulate (CC, bottom)) illustrate T1w/T2w (a) and R1 (b) slopes in gray and white matter (for all bundles see Supplementary Figures S6 and S7). Gray circles indicate gray matter values in each subject, colored circles represent white matter values in each subject, and the steepness of the lines indicate the slopes (rates of change relative to the infant's gestational age at the time of measurement in weeks), the two hemispheres are presented in different shades. **c,d** Relationship between the

slopes of T1w/t2w (c) and R1 (d) along white matter (WM) bundles and their gray matter (GM) targets (T1w/T2w: r^2=0.49, p-value=6.28e^{-5}; R1: r^2=0.45, p-value=0.0002). Each circle indicates a bundle, the two hemispheres are presented in different shades. Abbreviations: AF: Arcuate Fasciculus, ATR: Anterior Thalamic Radiation, CC: Cingulum Cingulate, CS: Cortico-Spinal Tract, FcMa: Forceps Major, FcMi: Forceps Minor, IFOF: Inferior Frontal Occipital Fasciculus, ILF: Inferior Longitudinal Fasciculus, MLF: Middle Longitudinal Fasciculus, OR: Optic Radiation, SLF: Superior Longitudinal Fasciculus, UNC: Uncinate Fasciculus, VOF: Ventral Occipital Fasciculus, pAF: Posterior Arcuate Fasciculus, L=left, R=right.

Specificity to regional vs global development - are the associations particularly different (across subjects) if you pick a different cortical target ROI for a fixed white matter bundle? i.e. Is it just individual differences in global T1 and T2w ratio as opposed to anything specific to the bundle?

Thank you - this is an interesting control analysis, which we have now implemented. As suggested, we randomly shuffled the bundle-target pairs 1000 times, omitting the true pairs, and then compared these "chance-level correlations" to our observed correlations. For both the mean values (Figure 2) and the slopes (Figure 3) the observed correlations were significantly higher than chance. We present these new analyses in supplementary figures S3 and S9 included below and report the finding in the results section as follows:

For the mean values, on page 4, lines 126-13 we write: "For both metrics, these observed correlations for the true bundle–target pairings were above the 95% confidence interval (CI) of chance-level correlations (95% CI of chance-level: r^2=0.14 for T1w/T2w and r^2=0.12 for R1) obtained by 1,000 iterations of correlating shuffled bundle-target pairings, omitting the true pairs (Supplementary Figure S3)."

For the slopes, on page 7, lines 230-233 we write: "For both metrics, these observed correlations for the true bundle–target pairings were above the 95% confidence intervals (CI) of chance-level correlations (95% CI of chance-level: r^2= 0.15 for T1w/T2w, r^2=0.14 for R1) obtained by 1,000 iterations of correlating shuffled bundle-target pairings, omitting the true pairs (Supplementary Figure S9)."

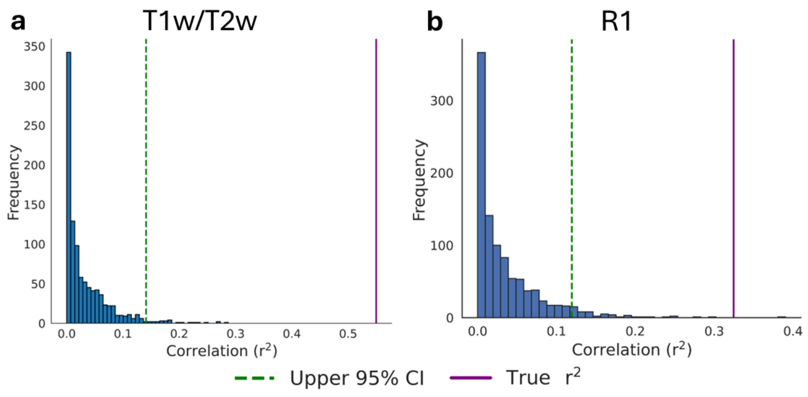

Figure S3: **Results of control analysis.** For this we shuffled bundle–target pairings to estimate chance-level correlations of mean T1w/T2w (a) and R1 (b) values across tissues. The histogram depicts chance correlations observed for 1000 iterations of shuffled bundle–target pairings that omitted the true pairings. The vertical dashed green line indicates the one-sided 95% upper bound of chance-level correlations, and the purple line indicates the observed correlation for the true bundle-target pairings. In both T1w/T2w and R1 the correlations for the true bundle-target pairings are above the 95% one-sided bound.

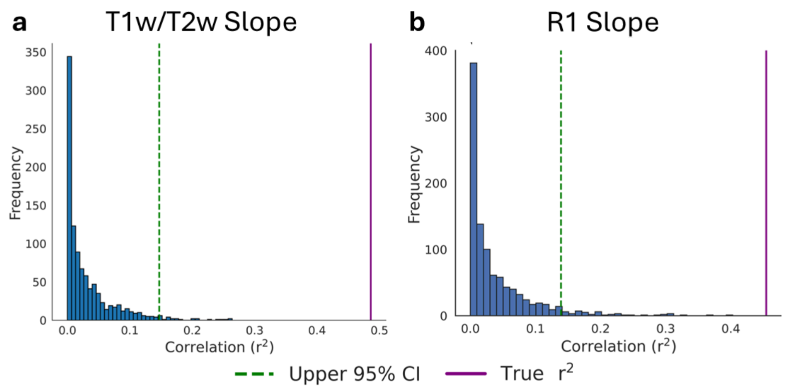

Figure S9: **Results of control analysis for slopes.** For this shuffled bundle–target pairings to estimate chance-level correlations of the slopes of T1w/T2w (a) and R1 (b) development across tissues. The histogram depicts chance correlations observed for 1000 iterations of shuffled bundle–target pairings that omitted the true pairings. The vertical dashed green line indicates the one-sided 95% upper bound of chance-level correlations, and the purple line indicates the observed correlation for the true bundle-target pairings. In both T1w/T2w and R1 the correlations for the true bundle-target pairings are above the 95% one-sided bound.

Use of raw Bayley scores - why were raw scores chosen? It seems like an unnecessary extra confound to add as these scores will scale on average with age of assessment. Did this test include age at scan / sex? Also were there sex differences?

Thank you for this comment. First, please note that we actually evaluated age-standardized rather than raw Bayley scores - this was incorrectly stated in the prior submission and we now corrected this oversight. We now also test the effects of birth age, scan age, and sex on the T1w/T2e coupling and found only the former two to be significant. These new results are presented in figure 4 included below. To address your concern, we performed additional model comparison analyses to formally test whether age at scan, gestational age at birth, or sex contributed to the prediction of motor outcomes beyond the T1w/T2w coupling (*r²*). Specifically, we compared a baseline model (*motor score* ~ *r²*) to three extended models that each included one additional covariate (scan age, birth age, or sex). None of the extended models provided a significant improvement in model fit relative to the baseline model.

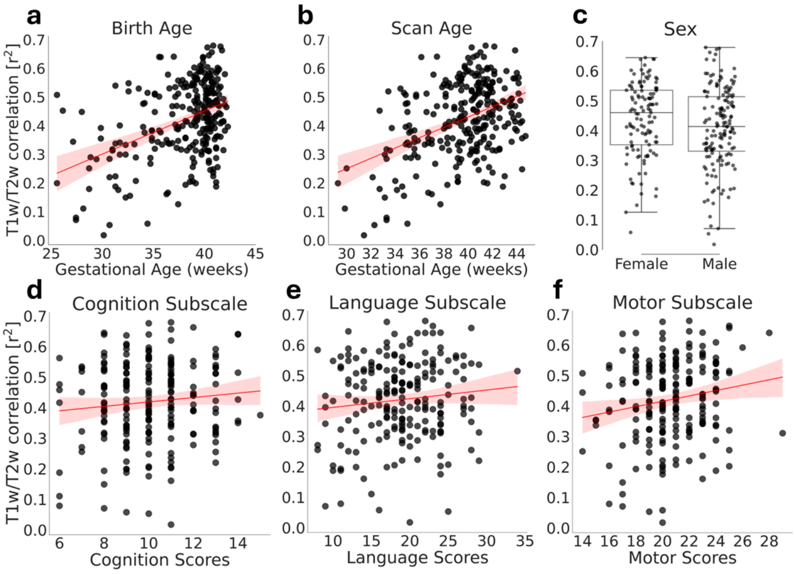

Figure 4: **Characterization of inter-individual differences in the T1w/T2w coupling and its relationship to behavior. a-c** Upper row relates T1w/T2w coupling across tissues to gestational age at birth (a, r^2=0.18, p-value=5.15e^{-13}), gestational age at scan (b, r^2=0.18, p-value=2.23e^{-13}), and sex (c, t=-1.88, p-value=0.06). **d-f** Bottom row relates the correlation of T1w/T2w in gray and white matter to the cognition (d; r^2=0.01, p-value=0.14), language (e; r^2=0.01, p-value=0.11), and motor (f;

r²=0.03, p-value=0.02) subscale of the Bayley-III. We found a relationship between the T1w/T2w coupling and motor skills, but not the other subscales. Each dot is a subject, red lines indicate linear regression lines, shaded regions indicate 95% confidence interval, boxes in c indicate the interquartile range (IQR, 25th–75th percentile), the line inside each box denotes the median and the whiskers extend to 1.5×IQR.

The corticospinal tract - this tract is a bit of an outlier to the others, in that it has substantially higher values in both GM and WM which does reflect it's higher myelin value. What happens to these associations when it is not included (e.g. figure 4).

Thank you. To evaluate this, we excluded the CST from the analysis presented in figure 2 (mean values) and figure 3 (slopes, formally figure 4). We find that the correlation of T1w/T2w mean values remains significant without the CS (r^2=0.21, p-value=0.02, see figure below) while the correlation of T1w/T2w slopes is not significant without the CS (r^2=0.12, p-value=0.10, see figure below). Please note however, that we also observe a significant correlation of R1 slopes and for R1 slopes the CS is neither an outlier nor does its removal impact the observed correlation (with CS: r^2=0.45, p-value=0.0002; without CS: r^2=0.43, p-value=0.0005). Moreover, we feel that it is a meaningful and somewhat expected finding that the CS is among the most developed bundles at birth, so we feel that is reasonable to include this bundle in the analysis.

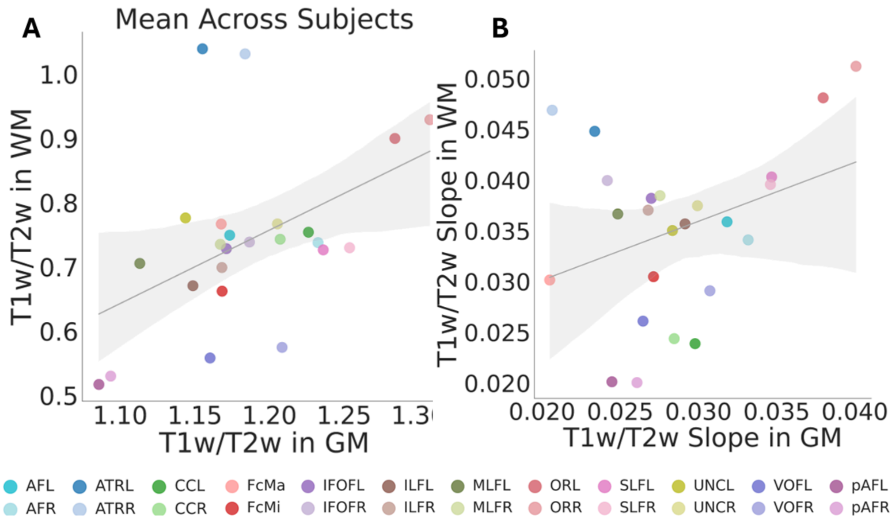

Relationship between T1w/T2w of white matter (WM) bundles and their respective gray matter (GM) targets without cortico-spinal tract (CS). Each dot is a bundle. (A) T1w/T2w values are averaged across all subjects (r^2=0.21, p-value=0.02). Relationship between the slope (rate of change) of T1w/T2w along white matter (WM) bundles and their gray matter (GM) targets (r^2 = 0.12, p-value = 0.10).

Reviewer #1

I would like to thank the authors for their thorough responses and for all the additional analyses performed in response to the earlier comments – they fully answered my concerns. I especially appreciate the inclusion of the parallel quantitative R1 dataset. It is reassuring to see that similar GM/WM coupling patterns emerge across both measures.

Thank you again for your feedback which we feel has enabled us to improve the manuscript.

Few very minor comments which I think can be taken care of at editing stage:

'We find that white matter and cortical myelin contents are tightly coupled: For both R1 and T1w/T2w values within each' : or f?
We corrected this.

'Further, in a longitudinal sub-sample, we find lower correlation of T1w/T2w across gray and white matter in preterm compared to full-term infants at birth..' one dot too many.
We corrected this.

'tissue response functions were computed': averaged over all subjects in the study? For the oldest subjects? Or you used individual responses?
Thank you. We have added this information. On page 17, lines 566-567 we now write: Using MRtrix3, first, tissue response functions for each subject were computed separately for white matter, gray matter, and CSF using the Dhollander algorithm.

'Are you perhaps referring to figure 3a-b?' Thank you, your clarification helps but I was actually referring to Fig 2. where you introduce: 'Mean correlation with T1w/T2w values averaged across subjects' I though somehow the correlation was meaned. To confirm, a,b are: Correlation of mean T1w/T2w bundle values averaged across subjects?
Yes, this is correct.

'If gestational age was the main predictor of motor skill, we would have expected that adding this factor would improve the model'. I would caution against this interpretation. If T1w/T2w coupling and GA are strongly correlated (as your results indicate), then adding GA to a model that already includes coupling would not necessarily improve model fit, even if GA were an important predictor. This simply reflects the fact that the second predictor contributes little unique variance when two variables share substantial variance. To assess whether coupling explains variability in motor outcomes beyond what can be attributed to GA, it may be more informative to directly compare models predicting motor outcome from coupling alone versus GA alone, or to examine partial correlations (outcome ~ coupling, controlling for GA).I am not disagreeing with the revised, more cautious conclusion -> this seems appropriate. I am including this reflection primarily for future reference regarding the modelling approach.
Thank you for your feedback, we agree and are happy to hear that the revised and more cautious conclusion seems appropriate.

Reviewer #2

I thank the authors for their corrections and clarifications to the previous review. I believe the changes that the authors have been made have substantially improved the manuscript. The addition of the local sample, including quantitative R1 and analyses between T1w/T2w and qR1, is informative. The manuscript and additions are well described. I have no further comments.

Thank you for your effort and helpful comments, which has enabled us to improve the manuscript.
